# Dendrimers, Dendrons, and the Dendritic State: Reflection on the Last Decade with Expected New Roles in Pharma, Medicine, and the Life Sciences

**DOI:** 10.3390/pharmaceutics16121530

**Published:** 2024-11-28

**Authors:** Donald A. Tomalia

**Affiliations:** 1 The National Dendrimer & Nanotechnology Center, NanoSynthons LLC, Mt. Pleasant, MI 48858, USA; donald.tomalia@gmail.com; Tel.: +1-989-317-3737; 2 Department of Chemistry, University of Pennsylvania, Philadelphia, PA 19104, USA; 3 Department of Physics, Virginia Commonwealth University, Richmond, VA 23284, USA

**Keywords:** dendrons, dendrimers, PAMAM, CNDP, dendrimersomes, Janus dendrimers, PEG alternatives, supramolecular dendrimers, amphiphilic dendrimers, mRNA delivery vectors

## Abstract

This perspective begins with an overview of the major impact that the dendron, dendrimer, and dendritic state (DDDS) discovery has made on traditional polymer science. The entire DDDS technology is underpinned by an unprecedented new polymerization strategy referred to as step-growth, amplification-controlled polymerization (SGACP). This new SGACP paradigm allows for routine polymerization of common monomers and organic materials into precise monodispersed, dendritic macromolecules (i.e., dendrons/dendrimers) with nanoscale sizes and structure-controlled features that match and rival discrete in vivo biopolymers such as proteins and nucleic acids (i.e., DNA, siRNA, mRNA, etc.). These dendritic architectures exhibit unprecedented new intrinsic properties widely recognized to define a new fourth major polymer architecture class, namely: Category (IV): dendrons, dendrimers, and random hyperbranched polymers after traditional categories: (I) linear, (II) cross-linked, and (III) simple-branched types. Historical confusion over the first examples of the structure confirmed and verified cascade, dendron, dendrimer, and arborol syntheses, while associated misuse of accepted dendritic terminology is also reviewed and clarified. The importance of classifying all dendrons and dendrimers based on branch cell symmetry and the significant role of critical nanoscale-design parameters (CNDPs) for optimizing dendritic products for pharma/nanomedicine applications with a focus on enhancing stealth, non-complement activation properties is presented. This is followed by an overview of the extraordinary growth observed for amphiphilic dendron/dendrimer syntheses and their self-assembly into dendritic supramolecular assemblies, as well as many unique applications demonstrated in pharma and nanomedicine, especially involving siRNA delivery and mRNA vaccine development. This perspective is concluded with optimistic expectations predicted for new dendron and dendrimer application roles in pharma, nanomedicine, and life sciences.

**In Memoriam of Professor Zofia Urbanczyk-Lipkowska**

There are no words to describe the personal emotions and sadness felt upon the loss of our dear friend, dendrimer colleague/scientist, and co-organizer of this 85th birthday-themed tribute, Professor Zofia Urbanczyk-Lipkowska (*deceased 22 January 2024). I knew Zofia for nearly 2 decades since being invited as a lecturer and guest of the Polish Academy of Sciences in 2008. At that time, Zofia and her husband Andrei hosted me and my wife Janet for a week in Warsaw. We soon became aware of their warm, generous, and kind-hearted manners, which quickly united us as friends and kindred spirits.

Both Janet and I have always been curious historians with a special interest in the culture and history of Poland. Andrei and Zofia shared the amazing history of Warsaw both pre- and post-WWII and the origin/evolution of the Polish Academy of Sciences, and they introduced us to several obscure but brilliant Polish artists whose paintings still adorn our home to this day. After Andrei’s passing, it was obvious that his loss was immense to Zofia as she immersed herself more deeply into her poly(peptide) science. However, she had an inexplicable new curiosity about the dendritic state and certain possibilities. It was through this mutual and shared interest in the dendritic state that I enjoyed such a long and enduring friendship with Zofia. She had always impressed me as a very hard-working, articulate, and outstanding poly(peptide) scientist with a certain hidden curiosity in dendrimer chemistry. That curiosity was soon revealed as Zofia shared a unique and unexpected scientific concept/dream that I considered to be both stunning and quite ambitious at the time. She proposed the synthesis and evaluation of specific poly(peptide) dendrimers (i.e., asymmetrical branch-cell type), which she felt would function as excellent structural mimics of certain natural membranolytic peptides known to exhibit unique anti-microbial activity (AMPs). Needless to say, with her typical, highly disciplined, articulate, and focused approach, she completed the synthesis of her novel dendrimer AMP mimics and determined they exhibited highly effective anti-microbial properties in record time [1]. This remarkable “scientific breakthrough”, based on the cleverly engineered key critical nanoscale-design parameters (CNDPs) of asymmetric branch-celled dendrimers, allowed Zofia to produce the first dendritic examples of low-toxicity, high-potency antimicrobial agents against clinical isolates of antibiotic-resistant *ESBL E. coli* strains. The discovery of these effective new dendritic agents against such multi-drug-resistant pathogens was widely recognized as a seminal breakthrough. This amazing work convinced me that Zofia was indeed an extraordinary, creative, and brilliant scientist who, without pause, earned my deepest respect and admiration.

It is with a “heavy and saddened heart” that I dedicate this modest but sincere “dendritic perspective” to Professor Zofia Urbanczyk-Lipkowska, a truly kind, generous, and sensitive human being, as well as a very creative and visionary scientist of the highest intellect. We are all very saddened by her loss, as well as knowing there are undoubtedly many more scientific visions and contributions that Zofia would have shared that now will remain incomplete. They could have enriched the dendrimer community and the sciences, as well as humanity in general. Zofia will truly be missed by all of us! 
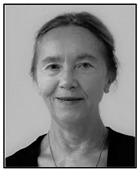
 Prof. Dr. Zofia Urbancyzyk-Lipkowska.

## 1. Prologue

The ultimate hope for all major new discoveries, such as Dendrimers, Dendrons, and the Dendritic State (DDDS), is that such a “scientific breakthrough” will serve humanity in some significant manner, as well as advance and translate into an important technology platform for enhancing the human condition by fulfilling many unmet needs in society, such as a more sustainable world food supply, better human health, increased longevity, or a generally improved quality of life worldwide.

My optimism for this hope and dream has been substantially heightened and reassured after reading the 12 outstanding contributions to the 85th birthday Dendrimer-themed special issue of *Pharmaceutics*. I am truly grateful to my dear friends and DDDS colleagues Prof. Zofia Urbanczyk-Lipkowska (Polish Academy of Science, Warsaw, Poland) and Prof. Anne-Marie Caminade (CNRS, Toulouse), who so skillfully mentored and organized this special-themed issue on Dendrimers. It was especially pleasing and exciting to note the progress and many new advancements described in these excellent scientific contributions coauthored by many highly recognized pioneers and colleagues from the DDDS community, including: V. Percec/D. Weissman [2], D. Astruc/C. Ornelas [3], A.-M. Caminade [4], R. Haag [5], R. Kannan [6], M.-C. Daniel [7], V. Cena [8], C. Kojima [9], J. Rodrigues, F. de la Mata [10], M.A. Munoz-Fernandez/E.R. Gillies/R. Gomez [11], S. Pricl [12], S. Calvo-Serrano, E. Matamoros, E. Perez-Inestrosa, C. Mayorga, M.J. Torres [13], and others. I am truly humbled, deeply honored, and very pleased by this extraordinary 85th birthday-themed tribute and will remain eternally grateful to each of you for your sincere and magnificent effort. This is an exceedingly thoughtful and meaningful gift to receive for my 85th birthday, 5 September, 2023, which I coincidentally shared with John Dalton on the occasion of his 257th birthday (born 5 September 1766).

## 2. Reflections on the Dendron, Dendrimer, and Dendritic State (DDDS) Discovery

### 2.1. Several Major Consequences/Advancements Resulting from the DDDS Discovery

In my humble and subjective opinion, several unexpected consequences resulting from the DDDS discovery that have benefited the major sciences and other disciplines should include the following:

(a) The DDDS discovery initiated a major paradigm shift in the polymer science area based on an unprecedented, multi-step, iterative polymerization strategy referred to as step-growth, amplification-controlled polymerization (i.e., SGACP).

Historically, polymer science has evolved into three major architectural categories; namely: (I) linear, (II) cross-linked, and (III) simple branched-type polymers. These traditional macromolecular architectures are derived from the assembly of monomers via two major polymerization or molecular weight-amplification strategies, namely: (1) chain-like addition polymerizations (i.e., free-radical, anionic, cationic propagations, etc.) or (2) step-growth, condensation polymerizations. In either case, one has very little control over absolute molecular weight homogeneity (i.e., polydispersity) and virtually no control over resulting macromolecular structures. As such, current polydispersity values for most commercial polymers generally range from 2–10. In contrast, these discrete dendron/dendrimer-type macromolecules exhibit very enhanced homogeneity features with polydispersity values of M_w_/M_n_ = 1.05–1.005 and allow substantial macromolecular structure control. These values are significantly better compared to the best known traditional living polymerization protocols. For example, as reported by Matyjaszewski, et al. [14], ATRP-type living free-radical polymerization results in M_w_/M_n_ = 1.2–1.3 and allows very little control over resulting macromolecular structures.

(b) The DDDS discovery has revealed a very versatile polymerization strategy for the synthesis of precise, amplification, and structure-controlled dendritic macromolecules using common organic monomers and reagents.

The resulting dendritic macromolecules possess monodispersity and structure control rivaling that are observed for natural biological polymers (e.g., proteins, enzymes, RNA, DNA, etc.). All original poly(L-lysine) dendrons (i.e., Denkewalter type) and poly(amidoamine) (PAMAM) dendrons and dendrimers (i.e., Tomalia type) are examples of unprecedented, new dendritic architectures and structures (i.e., dendrimers, dendrons, etc.) created via “step-growth, amplification-controlled polymerization” (i.e., SGACP) protocols. Using common organic reagents such as α-amino acids, ethylene diamine, acrylates, or acrylonitrile monomers with SGACP-type protocols has produced these unprecedented new dendritic macromolecular structures. The Denkewalter poly(L-lysine) (PLL dendron)- and Tomalia, poly(amidoamine) (PAMAM) dendrimer)-type structures represent the first examples of well-characterized/confirmed structures required for the historical verification of the DDDS discovery.

(c) Precise, amplification and structure-controlled dendritic macromolecules made possible with SGACP protocols offer extraordinary nanoscale property benefits to pharma, nanomedicine, and life sciences.

Dendrons and dendrimers allow unlimited access to vast libraries of precise, dendritic nanoscale, and macromolecular nanoparticles (i.e., NPs) that either mimic or possess equivalent dimensions observed for important natural polymers (i.e., proteins, nucleic acids, etc.), as well as many in vivo cellular or physiological compartments (i.e., 1–500 nm). Furthermore, the ability to engineer and modify these dendritic NPs as a function of their critical nanoscale parameters (CNDPs) (i.e., size, shape, surface chemistry, flexibility/rigidity, elemental composition, and architecture) is an invaluable feature for optimizing structures to fit specific application needs, especially for personalized pharma or nanomedicine.

(d) These unique dendritic macromolecules exhibit unprecedented new properties that define a major new polymer architecture class, namely: Category (IV): dendrimers, dendrons, and hyperbranched dendritic polymers.

**Figure 1:**
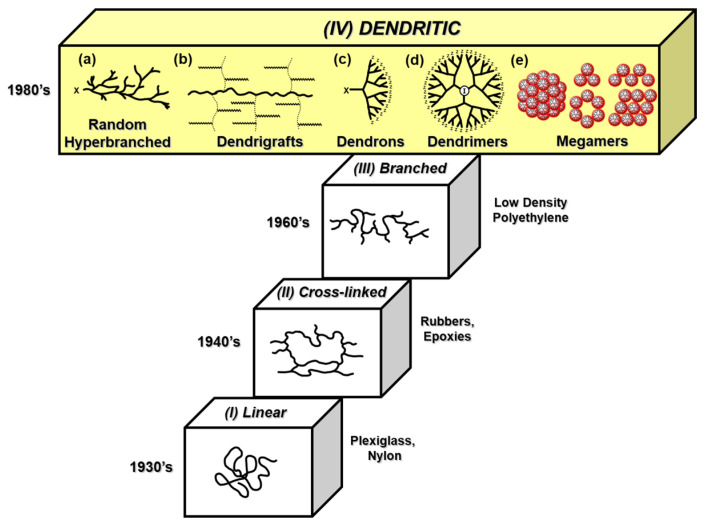
A chronology of the three major traditional polymer architectures with widely recognized applications, leading to the fourth new major Category (IV) of dendritic polymers: (**a**) random hyperbranched, (**b**) dendrigrafts, (**c**) dendrons, (**d**) dendrimers, and (**e**) megamers [15,16]. Reprinted and adapted with permission from Ref. [15]. Copyright 2002, John Wiley & Sons.

The iconic Berzelius hypothesis that states new molecular architecture creates new intrinsic properties has been fulfilled. A broad range of diverse, structure-controlled, dendritic macromolecules have been synthesized by these SGACP strategies and shown to exhibit a plethora of unexpected intrinsic properties, behavior, and associated phenomena. These new architecturally driven dendritic properties have been extensively characterized and demonstrated to fulfill many new applications, especially as active pharma ingredients (APIs), delivery vectors for targeted therapy, nucleic acid vaccine vectors, or in vivo bioimaging nano-devices in nanomedicine. These new architecturally driven dendritic properties have been extensively characterized and shown to exhibit unique utility in a wide variety of unprecedented applications. Collectively, these new dendritic structures/properties define the fourth-newest major polymer architecture category; namely: **Category (IV):** dendrimers, dendrons, hyperbranched and other dendritic polymers after traditional (I) linear, (II) cross-linked, and (III) simple-branched polymers as illustrated in Figure 1.

**Figure 2:**
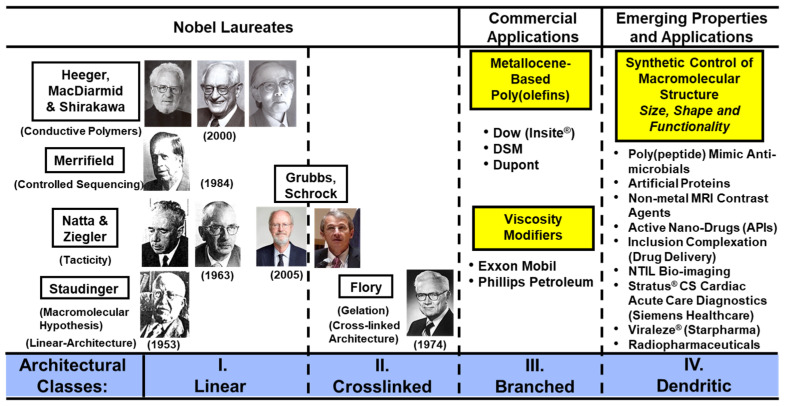
Nobel recognition, commercial applications, and new emerging properties for the four major polymer architectures. Reproduced with permission from Ref. [17]. Copyright 2013, Springer-Verlag Berlin Heidelberg.

The importance of these major polymer architecture categories is readily apparent by noting historical Nobel laureate recognition associated in the past for pioneering work involving the first three traditional major architecture classes, namely: Categories (I), (II), and (III) as illustrated in Figure 2.

### 2.2. Clarification of Historical Confusion Surrounding First Confirmed Examples of Authentic Non-Macromolecular and Macromolecular Dendrons and Dendrimers

A cursory survey of current literature and key archives (i.e., Wikipedia, etc.) reveals an alarming level of misinformation and confusion surrounding the general perception of the first historical examples of structure-confirmed and verified dendron/dendrimer syntheses. This confusion appears to result largely from a lack of accurate historical information and improper use of currently accepted IUPAC dendron/dendrimer terminology [18], as well as a plethora of incorrect or misused information. Such confusion has resulted in a distorted historical perspective concerning key contributions and specific examples that provided the first unequivocal confirmation of dendron and dendrimer structures considered to be absolutely critical and essential for the final acceptance of this seminal DDDS discovery.

A very small sampling of such examples is described below:

**Example** **1:** “Vögtle and his team (1978) reported the formation of the first non-skid chain-like and cascade-like molecules with the topology of the molecular cavity, which is considered the earliest dendritic polymer form. The term “hyperbranched polymer” was first coined by Kim and Webster in 1988 in reference to the synthesis of soluble hyperbranched polyphenylene. This term was later used to describe the structure of dendrimers” [19].

**Example** **2:** “Since the first dendrimer was reported in 1978 by Fritz Vögtle, dendrimer research has grown exponentially, from synthesis to application in the past four decades” [20].

**Example** **3:** “The term “dendrimer” was coined by the polymer chemist Donald Tomalia from the Greek “dendra” for tree and “meros” for part of at Dow Chemical in Midland, Michigan. Afterwards, in 1978, at the University of Bonn, Fritz Vögtle’s group synthesized dendrimers for the very first time when they tinkered with multi-functional branches ……” [21].

These excerpts and a brief account of this historical confusion merely document the “tip of the iceberg”. My greatest concern is that the historical integrity of the DDDS discovery for future generations of young dendritic scientists will be irretrievably lost or diminished, if not properly monitored and corrected. It is from this important perspective, as well as our common mission to strive for uncompromised integrity in all of our scientific endeavors by pursuing the “truth” (i.e., verified structure confirmations) and seeking accurate historical information with steadfast objectivity, that I share earlier critical DDDS research information made available to me by the Dow Chemical Company, Midland, MI, USA.

Recent access to officially released, confidential Dow Chemical Company dendrimer patent documents reveals a much clearer perspective concerning the actual synthesis and confirmation of first cascade structure examples, as reported by Vögtle, et al. in 1978 [22] (i.e., often erroneously referred to as the first examples of dendrimer structures). This important new Dow information, generated from 1990–1992, was in response to a final dendrimer/dendron patent rejection, wherein the 1978 Vögtle cascade publication was cited as prior art. Unfortunately, this important Vögtle reference was not discovered by Dow until the late 1980s. Widely recognized as a supramolecular scientist, Vögtle may have inadvertently caused the obscurity of this reference by comingling and introducing this extraordinary cascade structure/synthesis topic into one of his typical supramolecular articles. As such, all early literature searches failed to detect this important cascade synthesis reference. In fact, the only dendron/dendrimer-associated literature reference known to exist was the 1981 Denkewalter patent [23], which had been readily eliminated earlier as non-equivalent prior art.

As such, Dow expected all 8–10 Dow patent applications under examination in 1988 by the Japan National Patent Office (JNPO) would be granted without any difficulty. That said, a JNPO literature search was the first to reveal the obscure and undetected Vögtle cascade synthesis/structure reference, which had been missed by essentially all interested parties in this area [22]. This single reference was cited as prior art and led to final rejections of all Dow dendrimer patent applications in Japan.

In a final effort to overcome this negative JNPO decision, the Dow Chemical Co. Patent Department was directed to organize and fund a 1-year investigation entitled: cascade synthesis/structure reproducibility study. The objective of this effort was to determine if all cascade synthesis results, as well as reported cascade structures, could be reproduced and verified as reported by Vögtle in 1978. This Dow-funded investigation involved a neutral third party of highly qualified scientific experts who were directed to re-examine and confirm the reproducibility of cascade poly(propyleneimine) (PPI) structures as reported by Vögtle in the journal *Synthesis* [22]. Dow selected two well-recognized, organic synthesis experts, namely: Dr. Christoph Rickert, Ph.D. (ETH, Switzerland) and Dr. Lars Piehler, Ph.D. (Stanford University) for this 1-year funded investigation to determine the synthetic reproducibility of Vögtle’s cascade poly(propyleneimine) (PPI) structures. These Dow reproducibility study results are presented briefly below with more details available in the Supplementary Material Section.

**Scheme 1:**
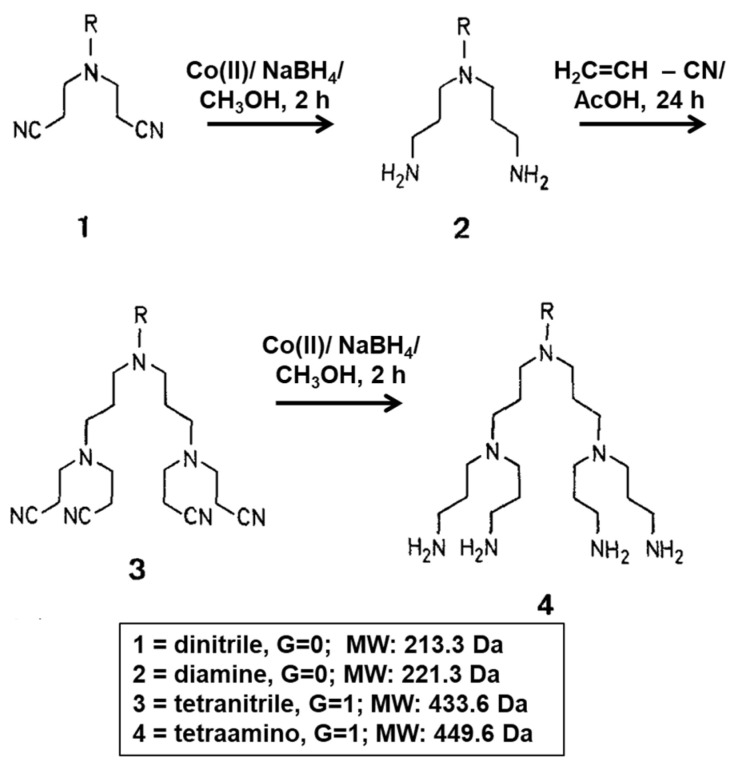
The divergent iterative synthesis of a cascade dendrimer precursor structure **3**, where: R = benzyl. Reprinted and adapted with permission from Ref. [22]. Copyright 1978, Georg Thieme Verlag KG.

As reported in this Dow-funded and officially certified cascade synthesis reproducibility study:

(1) There was “no evidence” that any multi-directional core (i.e., ethylenediamine core) derived true dendrimers could be synthesized or obtained by the Vögtle procedures published in 1978.

(2) The Dow study confirmed that only simple, low-molecular-weight di-directional benzylamine core-initiated dendritic-type structures could be synthesized and isolated according to Vögtle’s 1978 procedures. More specifically, only three low-molecular-weight benzylamine core-initiated dendritic cascade structures could be synthesized and confirmed. As described in Scheme 1, only the di-nitrile structure **1**; G = 0, MW: 213.3 Da; the di-amine structure **2**; G = 0; MW: 221.3 Da and the tetra-nitrile structure **3**; and G = 1.0, MW: 433.6 Da could be synthesized and confirmed. The structure **4**-tetra-amine, G = 1.0, MW: 439.6 Da could not be synthesized or detected using Vögtle’s 1978 procedures.

(3) All cascade structure product detection and confirmation were based on highly sensitive state-of-the-art mass spectrometry instrumentation (Electrospray Mass Spectrometer, Finnigan Model TSQ 700). It is very notable that no evidence could be obtained for the reproducibility of more complex multi-directional, dendrimer-based structures (i.e., structures 5b, 6b, 7b in the original Vögtle reference), resulting from a multifunctional initiator core such as ethylenediamine (EDA). (See as reported by Dr. C. Rickert and Dr. L Piehler in the certified documentation to the Dow Chemical Co. and disclosed in the Supplemental Section of this manuscript.)

(4) The essential absence of any meaningful product characterization data to support and confirm the cascade structures reported by Vögtle in 1978 was truly surprising. Absolutely no spectral data (i.e., FTIR, UV-vis, ^1^H, ^13^C-NMR) or chromatographic information (i.e., SEC, HPLC, etc.) were presented or reported to support these unprecedented cascade structures.

(5) The cascade product characterization and cascade structure confirmation were based solely on: (a) thin-layer chromatography (TLC) analyses, (b) standard C, H, N- elemental analyses, and (c) subjective interpretations of FAB mass spectrometry fragments. Vögtle reported that parent molecular ion masses for the cascade structures were either difficult or impossible to obtain, although these masses were detected routinely in the Dow study. Without any further activity in this cascade dendritic area from 1978–1993 (i.e.,~15 years), in 1993 [24], Vögtle mentioned that he had encountered substantial challenges with his 1978 cascade process that led to low cascade product yields, as well as characterization difficulties. These same Vögtle process difficulties were also noted by both Meijer, et al. [25] and Worner/Mülthaupt [26] later in 1993. Using their improved catalysts and revised processing conditions, Meijer and Worner/Mülhaupt independently reported obtaining very high cascade product yields via vastly improved protocols that proved suitable even for commercial production. For example, these new processing conditions allowed Worner/Mülhaupt to synthesize and isolate [(core: NH_3_ initiated] tri-directional, poly(propyleneimine) (PPI) dendrimer precursors (i.e., G = 0–1; Mwt.= 176–530 Da), as well as macromolecular dendrimers (i.e., G = 2–4; Mwt.= 1167–5133 Da).

In view of the failure to reproduce Vögtle’s results in the Dow-funded 1-year study, and the notable absence of critical product characterization/structure confirmation data in the 1978 Vögtle cascade synthesis article, the Japanese National Patent Office made an unprecedented decision to totally reverse their final patent-rejection decision. In fact, the JNPO accepted these Dow-funded reproducibility data as absolute evidence that no cascade, dendron, or dendrimer structures with MW > 433.6 Da had ever been synthesized or detected using the procedures as published by Vögtle in 1978. As a result, the Japanese National Patent Office officially granted all dendrimer and dendron patent claims to the Dow Chemical Company. This included all Tomalia PAMAM-type symmetrical branch-cell dendrimer and dendron structures possessing molecular weights higher than 433.6 Da (i.e., poly(propyleneimine) (PPI); tetra-nitrile; G = 1.0; MW = 433.6 Da).

In summary, the Dow Chemical Co.-funded “cascade reproducibility study” (1991–1992) provided important criteria for defining special terms such as true dendrimers and true dendrons. These terms were, accordingly, reserved for macromolecular, dendritic structures possessing MWts > 1000 Da. (i.e., generations > 2), whereas the term “cascade structures” was, accordingly, reserved for non-macromolecular, low-molecular-weight dendrimer precursor structures possessing MWts < 1000 Da.

**Figure 3:**
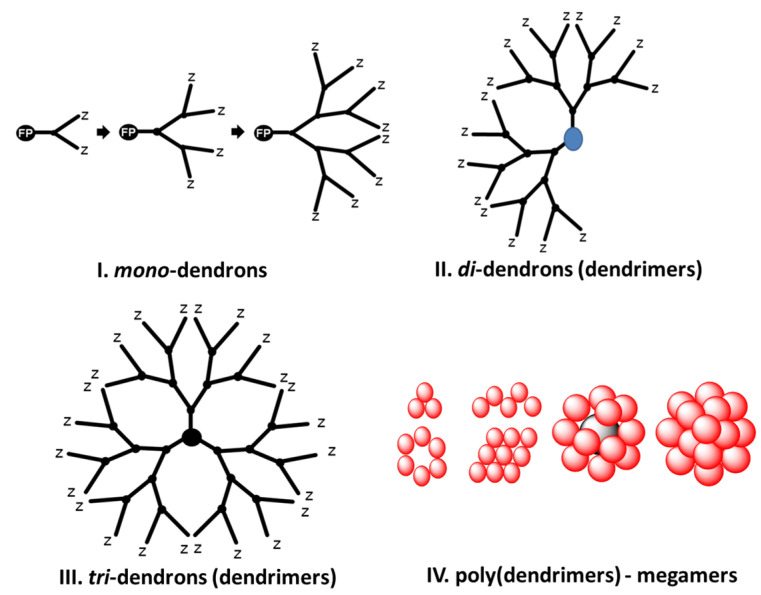
Structural designations for (**I**). monodendrons, (**II**). di-dendrons (dendrimers), (**III**). tri-dendrons (dendrimers), and (**IV**). poly(dendrimers)-megamers [28].

A brief review of currently accepted terminology and corresponding dendritic structures is described in Figure 3 [27].

For historical clarification, very little product-characterization data or meaningful structure-verification work was reported by Vögtle in 1978. It was not until 1983–1985 that critical confirmation and structure verification of various unprecedented cascade, arborols, and, especially, true dendron/dendrimer structures were reported.

The initial verification/confirmation of these novel synthetic dendron or dendrimer macromolecular structures appeared to be a very daunting challenge. In fact, the extraordinary proposal that these dendritic structures were expected to exhibit mathematically predictable and controlled MWts as a function of specific growth stages (i.e., generations) was considered to be virtually impossible by many. For that reason, and many others, the critical urgency for providing unequivocal and compelling dendritic structure confirmations was of the highest importance. It was very quickly realized that both macromolecular and small molecule-analytical protocols had to be articulately combined and integrated into a holistic, self-consistent confirmation of these novel dendritic structures in order to support a compelling and unequivocal verification of these unprecedented dendritic structures, as well as their non-traditional properties and behavior [29].

**Figure 4:**
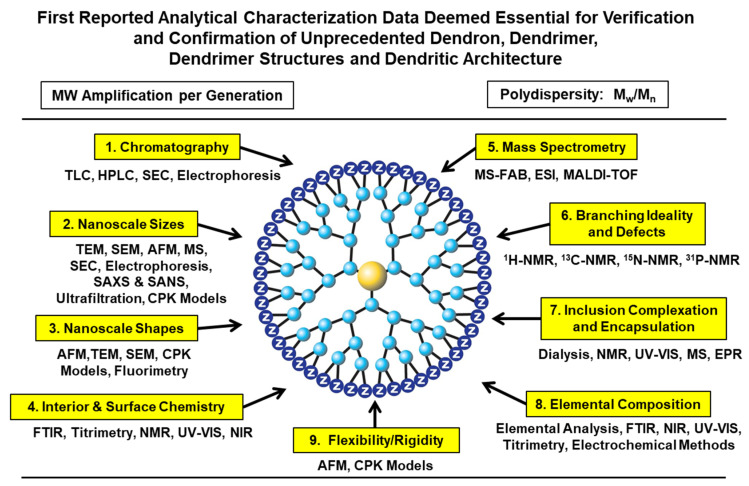
First reported analytical characterization data that led to confirmation of both symmetrical and unsymmetrical branch-cell dendrons, dendrimers, and megamers involved articulate documentation of critical nanoscale-design parameters (CNDPs) for these nanostructures.

A historical and annotated overview describing the first literature reports of analytical-characterization protocols that were required to verify these unprecedented dendritic structures and architectures is outlined below (see Figure 4) and discussed extensively elsewhere [30].

The first literature references and specific analytical protocols are described in Figure 4 below:

**MW Amplification/Generation and Poly(dispersity) (Mw/Mn):**

1. **Chromatography:** TLC [22], HPLC [31], SEC [31], electrophoresis (PAGE) [32]
2. **Nanoscale Sizes:** TEM [31,33], SEM [31], AFM [34,35], MS [31], SEC [31], electrophoresis (PAGE) [32], SAXS and SANS [36], Corey–Pauling–Koltun (CPK) models [31,37]
3. **Nanoscale Shapes:** AFM, TEM [31,33], SEM [31], fluorimetry [38], CPK models [31,37]
4. **Interior Surface Chemistry:** FTIR [31], titrimetry [31], ^1^H, ^13^NMR [31], UV-vis [31], NIR [39]
5. **Mass Spectrometry:** MS-FAB [31], ESI [40,41], MALDI-TOF [42]
6. **Branching Ideality and Defects:** ^1^H, NMR [31], ESI [40,41]
7. **Inclusion Complexation and Encapsulation:** Dialysis [43], NMR [31], UV-VIS [31], MS [31], EPR [38]
8. **Elemental Compositions:** C,H,N,O analyses [22], FTIR [31], NIR [39], UV-vis [31], titrimetry [31], electrochemical methods [31]
9. **Flexibility/Rigidity:** AFM [34,35], CPK models [31,37]

**Figure 5:**
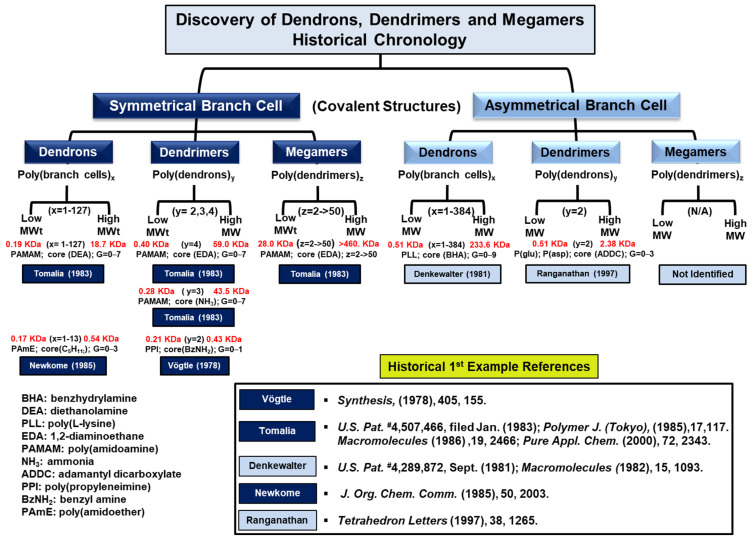
A chronology of historical first confirmed/verified examples of symmetrical and asymmetrical branch-cell dendrons, dendrimers, and megamers. First historical examples include: Vögtle [22], Tomalia [27,31,33,37,43], Denkewalter [23,50], Newkome [47], Ranganathan [48].

A chronology of these first confirmed examples is described briefly below and as illustrated in Figure 5.

- **F. Vögtle, et al.** (1978) [22]: the first confirmed, low-molecular-weight, “symmetrical branch-cell”, di-directional, (benzylamine core); oligo-(propyleneimine) cascade (OPI), “dendrimer precursor“ structures (i.e., G = 0–1.0; MWt: 213.3–433.6 Da) using iterative, divergent synthesis with in situ branch-cell formation protocols. The 1978 Vögtle cascade synthesis was shown by the Dow-funded study to yield only a low MWt (i.e., 433.6 Da), symmetrical branch-cell, oligo-(propyleneimine) (OPI) dendrimer precursor (i.e., G = 1.0; nitrile terminated). There was no detectable evidence that the reported tetra-directional core (i.e., [EDA core], PPI dendrimers) had ever been synthesized in 1978 by Vögtle procedures. Note: This confirmation and verification are based on the Dow reproducibility study (1992) since essential cascade structure characterization documented in Synthesis (1978) was either not reported, considered non-critical to the cascade structure confirmation, or was not reproducible.
- **R. Denkewalter, et al.** (1981) [23]: the first confirmed macromolecular, uni-directional, (dibenzhydryl amine core); poly(L-lysine) (PLL), “asymmetrical branch-cell” dendron structures (i.e., G = 0–9; MW: 511 Da–233,600 Da) using iterative, divergent synthesis with (protect–deprotect) protocols.
- **D. Tomalia, et al.** (1983) [31,44]: the first confirmed macromolecular, uni-directional (diethanolamine (DEA) core); poly(amidoamine) (PAMAM), “symmetrical branch-cell”, dendron structures (i.e., G = 0–7; MW: 445 Da~912 Da) using iterative, divergent synthesis, and (in situ branch-cell-formation) protocols.
- **D. Tomalia, et al.** (1983) [31]: the first confirmed macromolecular, tri-directional (NH_3_ core) poly(amidoamine) (PAMAM), ”symmetrical branch-cell” dendrimer structures (i.e., G = 0–7; MWts: 275 Da–43,451 Da) using iterative divergent synthesis and (in situ branch-cell-formation) protocols.
- **D. Tomalia, et al.** (1983) [31]: the first confirmed macromolecular, tetra-directional (EDA core) poly(amidoamine) (PAMAM),“symmetrical branch-cell” dendrimer structures (i.e., G = 0–7; MWts: 517 Da–116,493 Da) using iterative, divergent synthesis with (*in situ branch-cell-formation*) protocols.
- **D. Tomalia et al.** (1983) [31] (2000): the first confirmed examples of low-molecular-weight and high-molecular-weight, “symmetrical branch-cell” poly(dendrimers) referred to as “megamers”. Both random-structure megamers [27] and controlled-structure megamers referred to as core-shell tecto(dendrimers) [45,46] have been synthesized, characterized, and confirmed.
- **G. Newkome, et al.** (1985) [47]: the first confirmed, low-molecular-weight, uni-directional (pentane focal point core): poly(amidoether) (PAmE),”symmetrical branch-cell” dendron/arborol-type structures (i.e., G = 0–2; MW: 303 Da–1130 Da) using a six-step divergent synthesis and a preformed branch-cell protocol.
- **Ranganathan, et al.** (1997) [48]: the first confirmed, di-directional (1,3-adamantane carbonyl core); poly(glutamic acid) (Glu peptidyl), “asymmetrical branch-cell” dendrimers and di-directional, (1,3-adamantane carbonyl core); poly(aspartic acid) (Asp), “asymmetrical branch-cell” dendrimers, respectively. These dendrimers were each synthesized by first constructing uni-directional poly(Glu) dendrons (G = 1–3) or uni-directional oly(Asp) dendrons (G = 1–3), respectively. After deprotection of the (-NH_2_ end) focal point of the Glu or Asp dendrons, they were coupled to the 1,3-adamantane dicarbonyl chloride core in a convergent manner to obtain the respective Glu and Asp di-directional, asymmetrical branch-cell dendrimers.
- To our best knowledge, the first examples of asymmetrical branch-cell poly(dendrimer)-type megamers have not yet been synthesized or reported.

Earlier Dow–Tomalia evidence demonstrated the non-equivalency of Denkwalter’s asymmetrical branch-cell dendrons/dendrimers compared to the Dow symmetrical branch-cell dendrimer and dendron structures. This non-equivalency is based on highly differentiated intrinsic properties that result due to branch-cell symmetry in each case. As such, it is critical to divide all known dendrons and dendrimers into two distinctly separate categories, namely: Category (I): symmetrical branch cell and Category (II): asymmetrical branch-cell structures. This important issue of classifying all known dendrons and dendrimers according to their branch cell symmetry is discussed extensively elsewhere [49] and described briefly in Section 2.3.

Historically, the first critical characterization examples that ultimately led to confirmation, verification, and acceptance of all recognized dendron, dendrimer, and megamer structures were reported by five early contributors in the DDDS area, as noted in Figure 5.

That said, one of the most compelling characterization protocols leading to ultimate dendritic structure confirmation and acceptance was unarguably the invention of electrospray ionization (ESI) for mass spectroscopy by John Fenn in 1989 [51]. The application of these ESI protocols to demonstrate mathematically predictable, precise molecular weights as a function of generation was compelling to even the most severe critics of the DDDS discovery. Other equally compelling contributions that verified the DDDS concept should also include the convergent synthesis of dendrimers first reported by Fréchet/Hawker [52,53,54,55] and independently by Neenan, et al. [56,57], as well as the integration of main-row hetero-elements such as phosphorous, silicon, boron, germanium, or bismuth into a wide range of important dendrimer compositions as reviewed by Majoral/Caminade [58,59].

### 2.3. Why All Dendrons and Dendrimers Should Be Classified According to Branch Cell Symmetry

**Figure 6:**
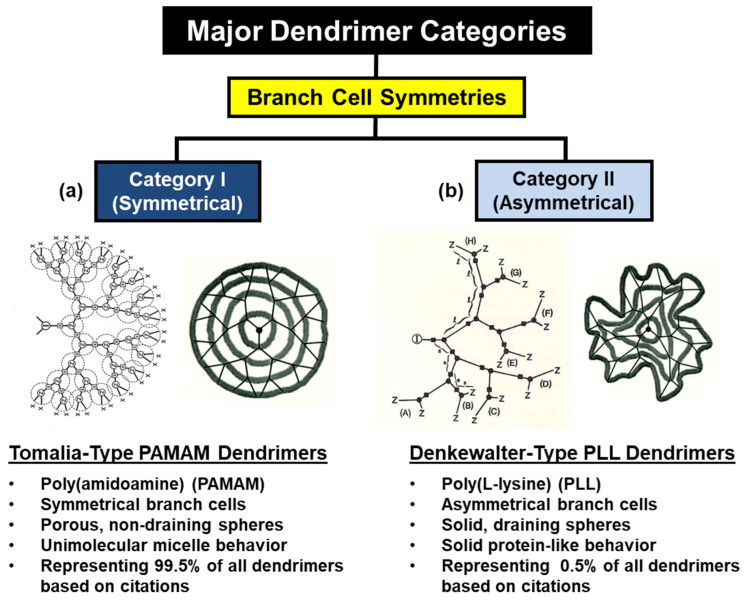
Classification of all dendrons and dendrimers according to branch-cell symmetry, namely: (**a**) Category (I): symmetrical branch cells represented by Tomalia-type PAMAM dendrimers, which are porous, non-draining spheroids that possess interior void space, exhibit guest–host inclusion complex-formation properties, and account for 99.5% of all literature citations. (**b**) Category (II): asymmetrical branch cells represented by Denkewalter-type PLL dendrimers, which are protein-like, solid draining spheres and account for 0.5% of all literature citations. Reprinted with permission from Ref. [49]. Copyright 2020, MDPI.

A little-known fact related to the DDDS discovery is that all early Dow Chemical Co. patent applications (1981–1984) for Tomalia/Dewald dendrimer inventions were rejected by the U.S. Patent Office. Based on the obscurity of the 1978 Vögtle cascade structure reference, extensive literature searches did not reveal any conflicting prior art to the 1979 Tomalia/Dewald Dow discovery of poly(amidoamine) (PAMAM) compositions. A portfolio of 8–10 Dow patent applications filed to protect these unprecedented dendritic architecture/compositions was expected to be granted and allowed without any difficulty. Receiving these patent rejections was truly stunning and devastating to the Dow Chemical Company, Midland, MI, USA. This unexpected U.S. Patent Office action was based solely on a single R. Denkewalter U.S. Patent #4,289,872 (1981) [23] to the Allied Chemical Co., wherein, the U.S. Patent Office had deemed the Denkewalter dendritic structures to be equivalent to those in the Dow applications. This disappointing event immediately initiated an extensive study to determine if a true equivalency between Denkewalter and Tomalia-type dendritic structures really existed. The ensuing assessment and comparison clearly confirmed a total absence of equivalency between the conflicting Denkewalter and Tomalia structures. Non-equivalency was unequivocal based on dramatically differentiated intrinsic, physico-chemical properties observed in each case. Although a casual examination of each highly branched structure type suggested equivalent dendritic architecture, it was soon revealed that “branch-cell symmetry” controlled and defined essentially all observed intrinsic dendrimer properties in each case. This included fundamental properties such as densities, refractive indices, interior solvent-filled void space, an ability to form (guest–host) inclusion complexes, the presentation of terminal surface groups, etc. [60,61,62,63], with several fundamental differences as noted below in Figure 6.

This critical demonstration of highly differentiated intrinsic properties observed for Category (I): symmetrical branch-cell dendrimers versus Category (II): asymmetrical branch-cell dendrimers clearly established the non-equivalency of Tomalia-type Category (I) and Denkewalter-type Category Type (II) dendrons/dendrimers, respectively. This differentiation led to the immediate allowance of all earlier Dow dendrimer patents, as well as literally dozens of subsequent patent applications in that area. These issues and details have been reviewed extensively elsewhere [49].

In retrospect, although this difference in dendron and dendrimer branch-cell symmetry (i.e., symmetrical vs. asymmetrical) appears to be minor, this subtle architecture difference truly confirms the power and importance of the Berzelius molecular architecture hypothesis, which states that any change in architecture at the molecular level, no matter how small or subtle, should be expected to produce entirely new intrinsic properties. Some of these intrinsic property differences are described in Figure 6.

This profound branch-cell symmetry effect has prompted an important and essential classification of all known dendrimer and dendron structures into two significant category types, namely: a major class of Category (I): symmetrical branch-cell structures and a minor group of Category (II): asymmetrical branch-cell structures. Denoting these major and minor branch-cell symmetry categories is based on the historical number of literature/patent citations documented for dendritic structures residing in Categories (I) and (II), respectively. A recent survey (i.e., patent and literature activity) of all known covalent dendrons and dendrimers has revealed a preponderance of activity (i.e., 99.5%) has focused on Category (I): symmetrical branch-cell dendrimers such as Tomalia and Newkome-type structures [64]. Meanwhile, Category (II): asymmetrical branch-cell dendrimers such as poly(peptide) and Denkewalter-type poly(L-lysine) dendrimers have received substantially less attention, but with recent indications that Category (II) activities may be increasing. In any case, we recommend the branch-cell symmetry classification of all covalent dendrons/dendrimers into Major Category (I) or Minor Category (II), respectively, as described in Figure 6.

That said, dramatically enhanced interest and activity have been observed in the asymmetrical branch-cell dendrimer area (i.e., poly(peptide) dendrimers). More specifically, this activity has been inspired and catalyzed based on recent new advancements in synthesis [64,65] and discovery of new intrinsic properties [66], as well as exciting new developments in a variety of medical applications, including dendritic mimics of multi-drug resistant anti-microbial peptides (AMPs) [1,67,68,69], anti-bacterial [70], anti-fungal, and anti-viral agents [64].

### 2.4. The Important Role of Critical Nanoscale-Design Parameters (CNDPs)

A brief survey of the 12 outstanding articles contributed to the current 85th birthday-themed issue on DDDS (2023-2024) has revealed the following interesting trends based on topics covered and discussed in Table 1.

Essentially every contributed article to this current special topics issue touches on some aspect of dendron and dendrimer-related critical nanoscale-design parameters (CNDPs) followed by targeted/drug delivery, amphiphilic/supramolecular assemblies, heteroatom-containing and dendritic APIs, etc.

**Figure 7:**
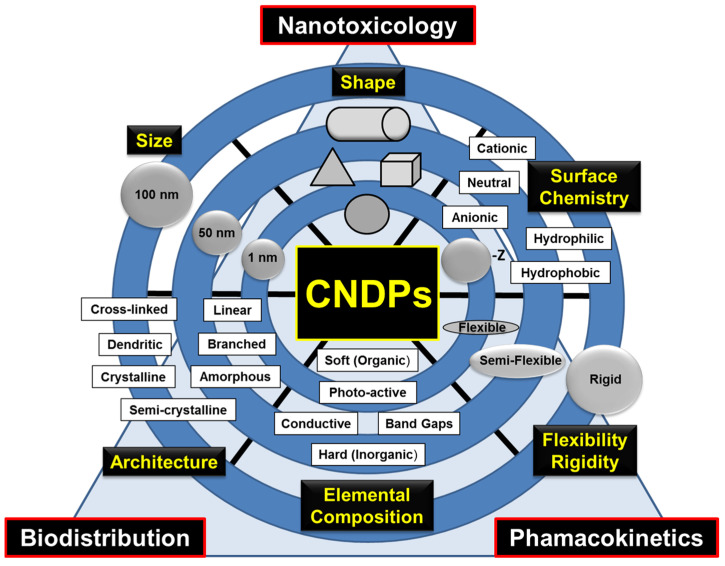
Six critical nanoscale-design parameters (CNDPs) and their important role in determining the nano-toxicity, pharmacokinetics, and biodistribution of well-defined, hard/soft nanoparticles (NPs) used in drug delivery and nanomedicine applications [28,63,72,73].

Intrinsic nanoparticle property patterns based on the six CNDPs, such as size, shape, surface chemistry, flexibility/rigidity, architecture, and elemental compositions, have provided powerful and predictive guides for optimizing nanoparticle features. This concept was introduced as early as 2010 for dendrimers and all well-defined hard/soft nanoparticles (NPs) [63,71]. These special features are required for many nanomedicine applications, including their use as APIs, nanoscale vectors for MRI contrast agents, solubilization and encapsulation of drugs, or as site-specific, targeting agents for the delivery of small drugs/nucleic acids. Using these CNDP principles, the optimization of many pharmacokinetic and biodistribution, as well as excretion and nano-toxicology, properties have been demonstrated for a wide range of well-defined hard and soft nanoparticle platforms [71] as illustrated in Figure 7. The importance of exploiting CNDPs and their widespread use in nanomedicine has been discussed extensively elsewhere [72,73,74].

A specific and important application of these CNDP principles has recently been demonstrated by the simple modification of PAMAM dendrimer terminal surface moieties to render them (a) more bioavailable, (b) non-toxic, (c) NTIL fluorescent, and (d) stealth to innate immuno-responses.

**Figure 8:**
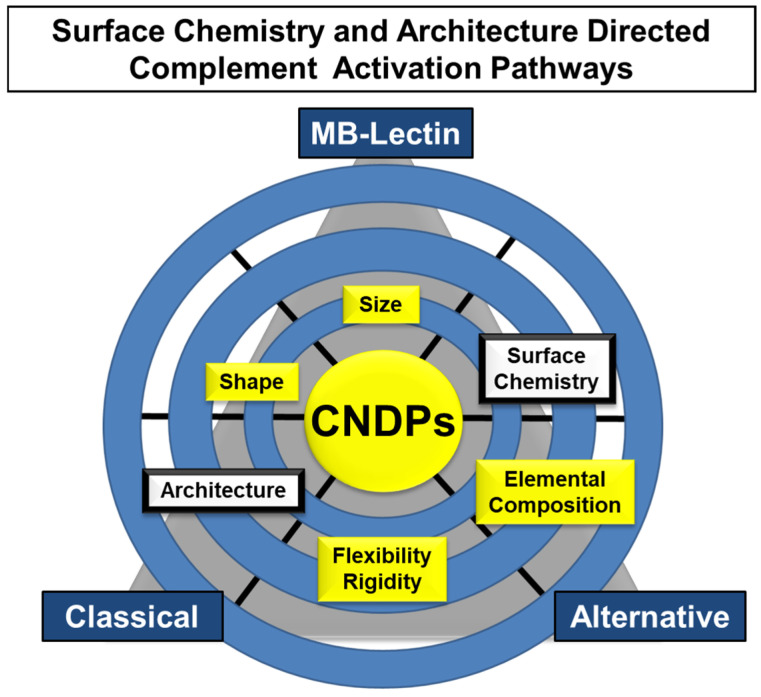
Six critical nanoscale-design parameters (CNDPs) and the important role that “surface chemistry” plays in determining stealth features and non-complementary activation properties for well-defined nanoparticles such as dendrimers.

Currently, one of the greatest challenges confronting nanomedicine is the in vivo delivery of therapeutic drug levels to disease sites with minimal collateral exposure to healthy tissue while avoiding innate immune system recognition [75]. Essentially all living organisms have evolved and survived by developing innate immune recognition strategies for eliminating deleterious pathogens or other foreign objects that may interfere with normal physiological processes. These innate immunological defenses involve both humoral and cellular recognition mechanisms and are generally referred to as the complement system. The complement system consists of specific soluble plasma proteins, as well as membrane-bound protein regulators/receptors that may be recruited to activate a cascade of reactions directed at the elimination of a pathogen/foreign object from the organism. The complement system utilizes a diverse repertoire of “pattern-recognition strategies” to identify dangerous pathogen signals. Activation signals associated with pathogens may include conserved microbial cell wall components, carbohydrate/protein, or other poly(hydroxyl)motifs. In general, any surface chemistry/moieties/features leading to serum protein adsorption (i.e., opsonization) will be expected to produce a strong immune response through complement activation. The complement system is an integral component of the innate immune system. It recognizes intruder danger signals primarily through pattern recognition, a process mediated by complement-sensing molecules such as IgG, IgM, and IgA class antibodies, C1q, mannose-binding lectin (MBL), ficolins, and properdin [75]. These recognition events are programmed to trigger various complementary cascade or activation sequences, which are generally recognized to follow any of three major courses, referred to as (1) the classical, (2) lectin, or (3) alternate-activation pathways. Inadvertent activation of the complement system will elicit acute reactions and pro-inflammatory responses, including anaphylaxis shock, which may be fatal. These complementary activation cascades are often observed following infusion or in vivo use of many nanomedicines, nanoparticles, or polymeric structures in animals and humans [75,76,77]. There is now growing evidence that these same nanoparticle-based CNDP principles may be applied to NP engineering strategies for avoiding in vivo innate immune recognition and complement activation (Figure 8).

**Scheme 2:**
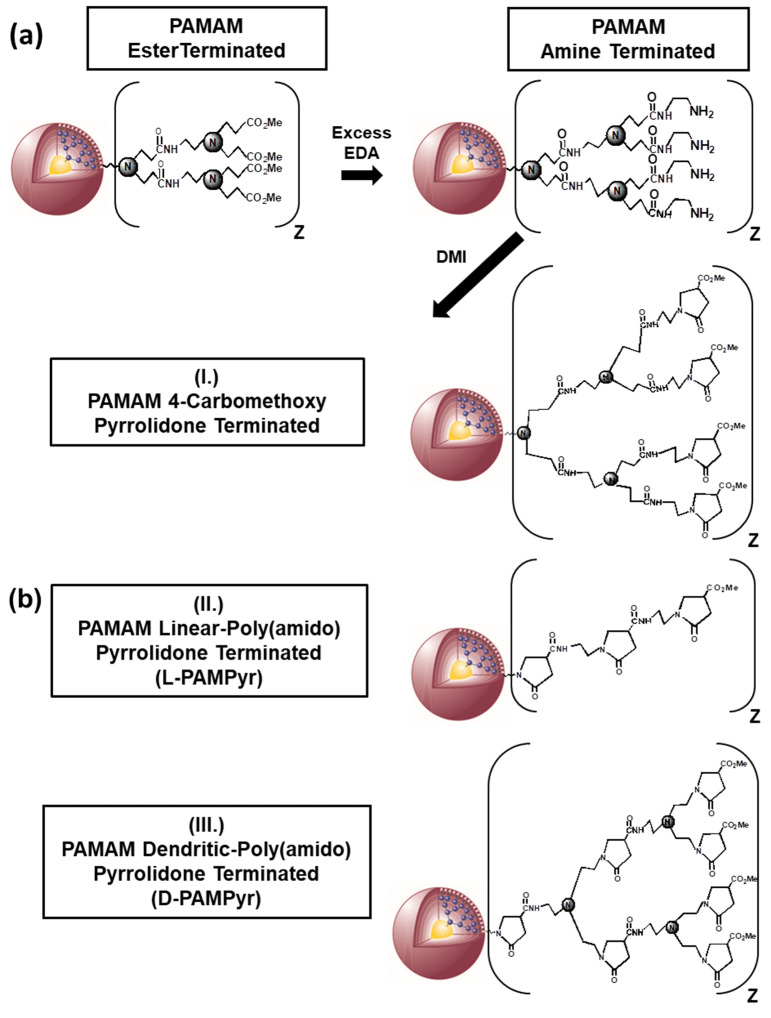
(**a**) Unique modifications of amine-terminated PAMAM dendrimers with dimethyl itaconate (DMI) to produce stealth-like, non-complementary activating properties, (**b**) other examples of PYRROLIDONylation involving the modification of amine or pyrrolidone-terminated PAMAM dendrimers [78].

A simple CNDP-surface chemistry modification of an important class of nanoparticle vectors such as PAMAM dendrimers has been shown to produce “stealth-like features“ and exhibit non-complementary activation properties analogous to those observed for traditional PEGylated drugs and products. More specifically, the reaction of amine-terminated PAMAM dendrimers with dimethyl itaconate provides essentially quantitative conversions into 4-carbomethoxy pyrrolidone terminated dendrimers as illustrated in Scheme 2a. Considered to be analogous to PEGylation, this unique pyrrolidone modification of amine-terminated dendrimers is referred to as PYRROLIDONylation. Other demonstrated examples of PYRROLIDONylation by modification of dendrimer surface chemistry are illustrated in Scheme 2b. PYRROLIDONylation represents an alternative strategy to PEGylation and produces unprecedented examples of unique nanoscale materials that exhibit amazing non-complementary activation properties as predicted by Tomalia et al. [78], subsequently reported by Christensen/Klajnert–Maculewicz [79] and later demonstrated by Moghimi, et al. [80]. These “PYRROLIDONylation” products should be viewed as important and cost-effective alternatives to PEGylated products, which are currently under scrutiny due to recent confirmation that the “innate immune system” recognizes and produces detectable immuno-responses (i.e., antibodies) after repeated usage [81,82,83].

### 2.5. A Decade (2012–2022) of Expansion and Growth for Supramolecular Dendritic Structures (i.e., Dendrimersomes, Nano-Micelles, Dendri-Micelles, Supramolecular Dendrimers, etc.)

In addition to significant growth noted for traditional covalent-bonded dendritic structures over the past decade, explosive growth and expansion have occurred with supramolecular, non-bonded, dendritic structures and architecture. Many novel amphiphilic and Janus-type dendrons and dendrimers have been described by new terms/terminology (i.e., hybrid and supramolecular dendrimers). They have been self-assembled into a wide range of new dendritic supramolecular assemblies that exhibit unique and unprecedented properties [84,85,86].

The first peer-reviewed publication describing intrinsic supramolecular features of dendrimers appeared in 1988 [87,88]. Subsequent supramolecular perspectives of the “dendritic state” began to emerge as early as 1995. At that time, both Meijer, et al. [89] and Percec, et al. [90] recognized that amphiphilic dendrimeric entities did not self-assemble according to traditional Israelachvili-type amphiphilic surfactant rules [91] but produced new, unexpected dendritic, micelle-like assemblies. These unique, unprecedented dendritic self-assemblies appeared to result from the significantly larger hydrophilic, dendritic head component compared to the traditional dimensions of the hydrophobic linear tail components. Concurrently, Zimmerman, et al. [92] reported the first self-assembly of focal-point functionalized dendrons possessing suitable “hydrogen-bonding” features. These were the first examples of true supramolecular dendrimers. This seminal event was soon followed by the assembly of carboxylic acid functionalized focal-point dendrons around metal cations according to Fréchet, et al. [93], as well as the assembly of excess carboxylic acid-terminated shell reagents around a limited amount of amine-terminated PAMAM dendrimer to produce charge-neutralized carboxylic acid terminated core-shell tecto(dendrimer) precursors, which were then converted into their covalent form with carbodiimide reagents [45].

A variety of supramolecular issues related to dendrimers and dendrons included “all-or-nothing reactivity” with hydrophobes as a signature for de Gennes dense packing, for multivalent supramolecular dendrimer-drug interactions [94], aggregation of amphiphilic PAMAM dendrimers G = 0–3 [95], the mimicry of classical regular and inverse micelles, etc., while “guest–host” encapsulation properties were reviewed extensively elsewhere [95,96,97].

#### 2.5.1. Amphiphilic Janus Dendrimers: Self Assembly into Dendrimersomes

**Figure 9:**
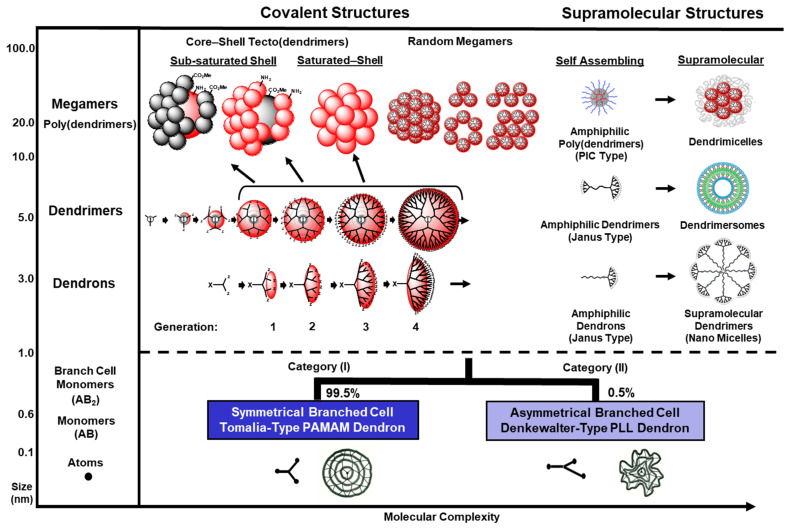
Various dendrons, dendrimers, and megamers as a function of known covalent and supramolecular structures. Proposed division of all known dendrons/dendrimers according to branch-cell symmetry, namely: the major Category (I) possessing symmetrical branch cells and the minor Category (II) possessing asymmetrical branch-cell structures.

**Figure 10:**
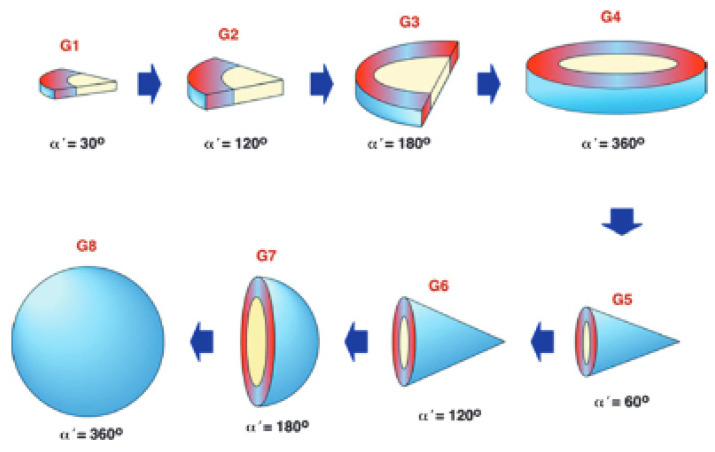
Hierarchical control of self-assembly via molecular solid angle. Reprinted with permission from Ref. [102]. Copyright 2000, American Chemical Society.

Throughout the timeframe 2009–2014, a more intense interest began to emerge with a specific focus on dendritic amphiphilic entities, such as the first examples of Janus dendrimers, which appeared in a patent [98], amphiphilic Janus-type PAMAM dendrons, as well as other modified dendrimers (Figure 9). It involved a number of pioneering scientists, including Percec, Peng, Velder, et al. and others. It began with the pioneering work of Percec, et al. in 2009 [99,100,101,102,103]. This seminal work demonstrated that predictable nano-periodic, dendron-shape changes occurred as a function of the generation level. These predictable and quantized nano-patterns of hierarchical shape changes were manifested as unique dendron morphologies. These unique morphologies obtained as a function of the generation were readily defined as solid-angle dendron projections (α′) when projected onto a plane (Figure 10). The α′ angle is defined as the solid-angle projection of the dendron onto a plane and can be determined in all cases according to (α′) = 360/μ, wherein μ is the number of dendrons in a specific column stratum or supramolecular sphere. Increasing the branching of a tapered-like dendron via a sequence change or an increase in generation number increases the α′ and the fraction of the disk occupied in a columnar self-assembly. At a certain threshold, only disks are formed. Above this threshold, further branching results in congestion-induced deformation of the disk into a conical segment with diminished α′. Beyond this point, enhanced branching increases (α′), as well as the fraction of a sphere formed. These transformations are illustrated as a function of generation levels 1–7. Ultimately, an unimolecular spheroid should result at G = 8, as shown in Figure 10 [102].

Within the context of this quantized and predictable behavior, Percec et al. [99] systematically investigated the effect of the generation number on the self-assembly of various amphiphilic dendrons. Using X-ray diffraction studies [102], it was revealed that G = 1 and G = 2 dendrons tended to form 2-D, flattened disc-like shapes that self-assembled into cylindrical columns. On the other hand, G = 3 dendrons underwent a congestion-induced shape change resembling a 3-D fragment of a sphere and tended to form spherical aggregations within a cubic lattice. In essence, it was confirmed by X-ray analysis that congestion-induced shape changes to produce a spheroid-like morphology should be expected to occur as a function of generation level. This was an amazingly concise alternative corroboration of a similar generation-dependent, congestion-induced shape change observed by Tomalia/Goddard et al. in 1989 [62]. Many of these first principles were subsequently applied by Percec et al. [99] to gain a deeper understanding of these nanoscale self-assemblies. They were then used in the development of extensive libraries describing the self-assembly modes and ultimate 3-D lattices formed by these amphiphilic dendrons. Extensive X-ray characterization and retro analyses of these 3-D lattice libraries have produced quantitative roadmaps for understanding critical dendron-structural parameters that direct these self-assembly patterns. This information now allows for an accurate a-priori prediction of final self-assembled structures one would expect based on certain critical nanoscale-design parameters. It has now been shown that these controlled CNDPs such as size, shape, surface chemistry, and flexibility [100] may be analyzed in the primary amphiphilic dendrimer structures and used for these predictions [100]. Historically, this was the first demonstrated example and proof that one could predict the final supramolecular-type assembly that would form with an accuracy of >85% [100]. These predictions were based on known dendrimer nano-periodic property patterns and the specific CNDP design of the Janus dendron. These results actually suggested and supported a concept for creating a Mendeleev-like nano-periodic table for well-defined dendron and dendrimer nanoparticles.

This seminal work was discussed in great detail with V. Percec on the occasion of a historical *IDS-6* dinner in Stockholm, Sweden (2009). This revelation clearly connected with my long-term interest in defining a unifying nano-periodic property concept for explaining the many pervasive nano-periodic property patterns that have been observed for discrete quantized nanoparticles that appear to mimic atoms, such as dendrimers, dendrons, etc. This Percec work appeared to be a “demonstration of concept” for a unified nano-periodic concept as proposed elsewhere in collaboration with physicists such as Prof. Shiv Khanna (VCU), et al. for other super atoms (i.e., nanoscale atom mimics) [104,105,106]. This seminal work by Percec clearly demonstrated the remarkable predictive power with >85% accuracy and potential implications for Percec’s first predictive tables for the self-assembly of amphiphilic Janus dendrimers. Historically, these data may have fulfilled/validated our proposed nano-periodic concept for unifying well-defined quantized nanoparticles and represent the first example of a Mendeleev-like nano-periodic table. These issues have been discussed and described extensively elsewhere [17,104,105,106].

**Figure 11:**
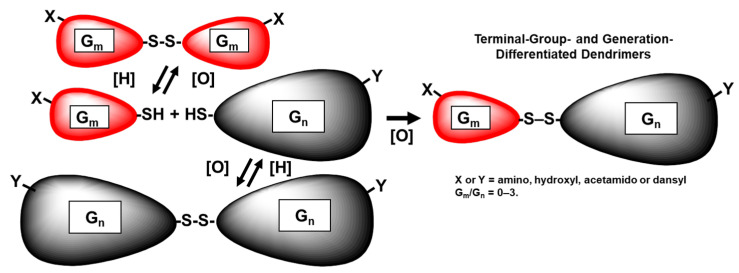
The first example of a Janus dendrimer was obtained by reduction and re-oxidation of two differentiated (i.e., surface and generation differentiated) cystamine core PAMAM dendrimers. Reprinted with permission from Ref. [16]. Copyright 2005, Elsevier [107].

A preponderance of the earlier pioneering dendrimer work by Percec, et al. [99] in the 1990s had been focused on the self-assembly of amphiphilic dendrons. That effort subsequently evolved into the supramolecular assembly of amphiphilic dendrimers currently referred to as Janus dendrimers [19]. A Janus-type dendrimer is formed by linking two chemically differentiated features or components (i.e., a hydrophobic and hydrophilic domain) into a single dendrimer structure. Some of the earliest examples of surface-differentiated Janus dendrimers were reported by Hawker/Fréchet [54], as well as by Tomalia, et al. [107,108,109], as illustrated in Figure 11 below.

**Figure 12:**
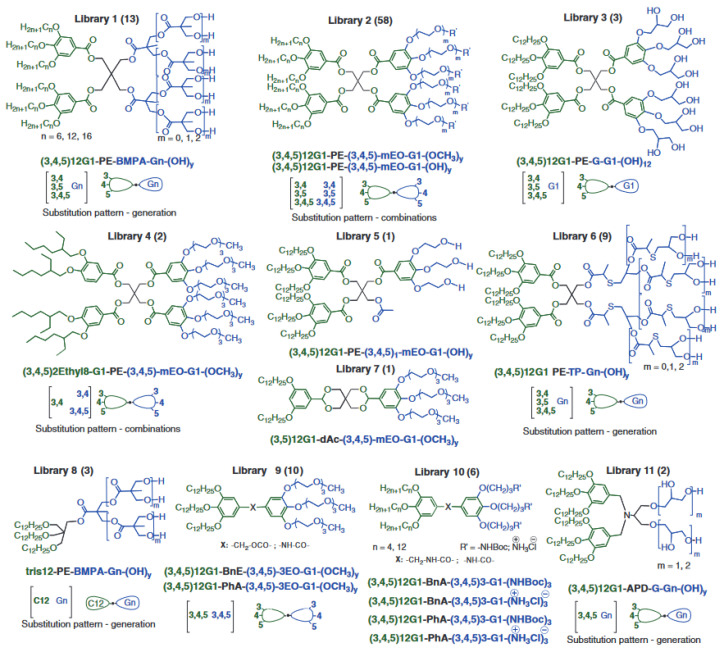
Various libraries of Percec-type Janus dendrimers suitable for self-assembly into dendrimersomes. Reprinted with permission of Ref. [109]. Copyright 2010, The American Association of the Advancement of Science.

As early as 2010, Percec, et al. [109] reported the synthesis of a broad class of amphiphilic Janus dendrimers with typical structures, such as those illustrated in Figure 12. Over 11 distinct libraries consisting of more than 107 uncharged and cationically charged dendrimers were examined [109]. By simply injecting an ethanolic solution of Janus dendrimers into water, they obtained very uniform, robust populations of bilayer vesicle assemblies reminiscent of liposomes. These new unprecedented Janus dendrimer self-assemblies, which are in essence megameric assemblies (i.e., poly(dendrimers)) that have been coined—dendrimersomes and are as shown in Figure 12.

**Figure 13:**
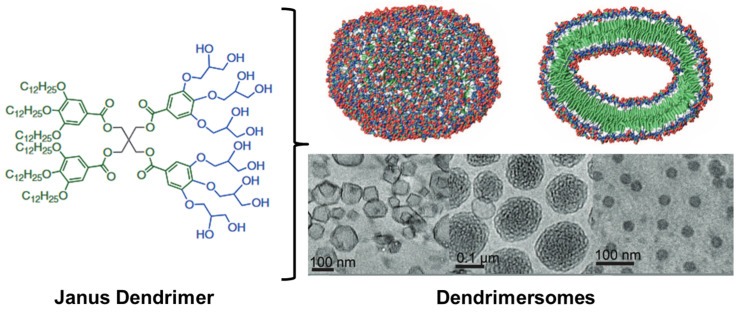
Self-assembly of a Percec-type Janus dendrimer into a dendrimersome. Reprinted and adapted with permission of Ref. [109]. Copyright 2010, The American Association of the Advancement of Science.

It is readily apparent that these Janus dendrimers are powerful, structure-directing amphiphiles that exhibit greater versatility than simple lipids, surfactants, or block copolymers in their ability to produce uniform, robust, and highly predictable functional assemblies Figure 13. Based on specific dendrimersome CNDP features and associated principles, Percec was able to predict the type of supramolecular dendrimersome self-assembly structure that would result in an accuracy of >88%. This suggests the strong possibility for evolving a variety of unprecedented and predictive Mendeleev-like nano-periodic tables [101].

**Figure 14:**
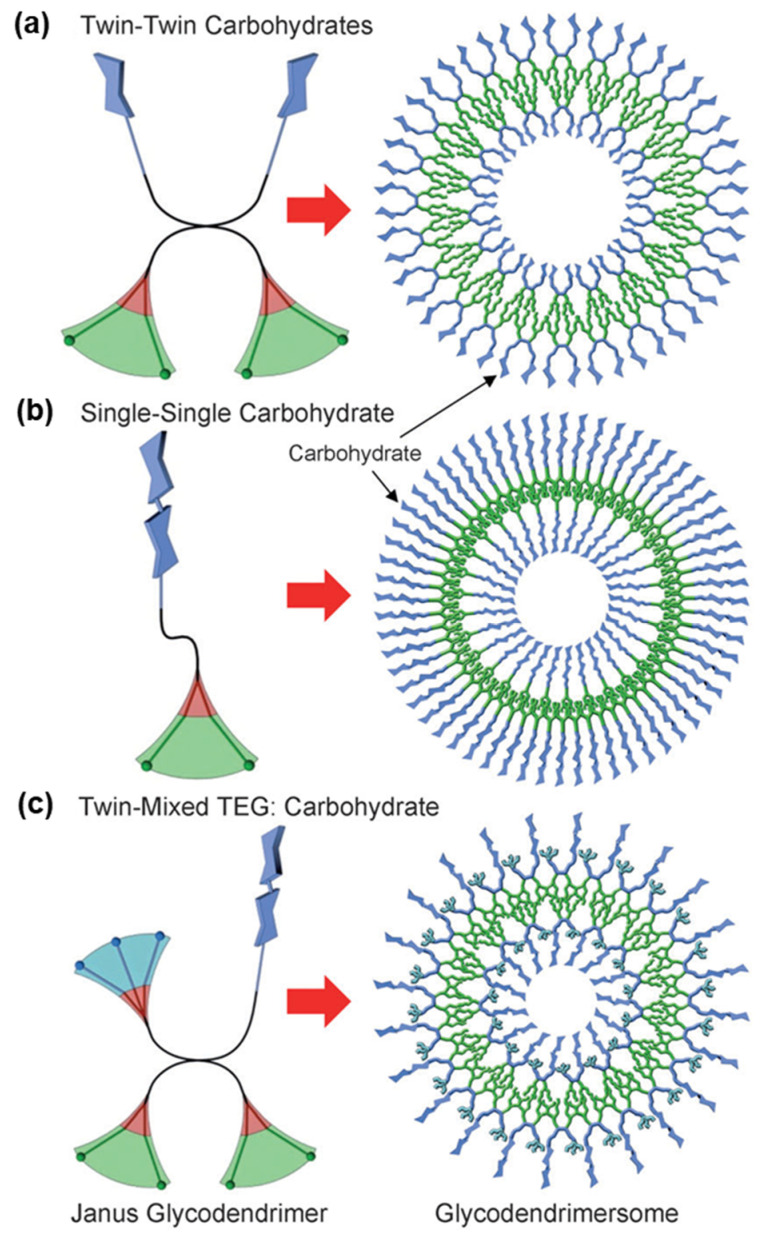
Various “twin–twin”, single–single”, and “twin–mixed” carbohydrate presenting structures and libraries. Color code: hydrophilic blue, hydrophobic green, aromatic red. Reprinted with permission from Ref. [110]. Copyright 2014, John Wiley & Sons.

**Figure 15:**
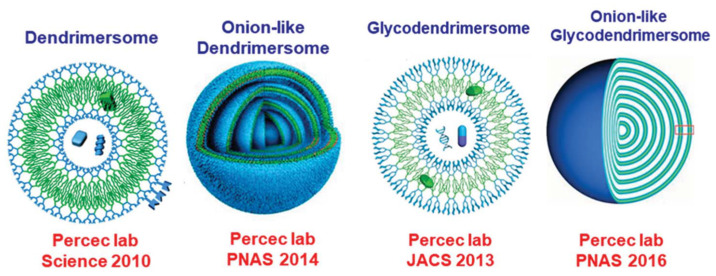
Four major categories of Percec-type dendrimersomes. Reprinted and adapted with permission from [111]. Copyright 2023, MDPI.

A wide range of diverse Janus-type dendrimer structures have been synthesized. A small sampling of these Janus-type dendrimer libraries reveals that unique structures such as “twin–twin”, “single–single”, and “twin–mixed” carbohydrate-presenting entities have been made, as illustrated in Figure 14. Depending on the structure of the Janus dendrimer precursor, at least four major dendrimersome topologies were obtained, as illustrated in Figure 15.

These unique dendrimersome assemblies have been recently demonstrated to play a critical role in new simplified, one-component protocols for the delivery of mRNA in future vaccines, as described later.

#### 2.5.2. Amphiphilic, Janus Dendrons: Self-Assembly into Supramolecular Dendrimers (i.e., Dendrimeric Nano-Micelles)

**Figure 16:**
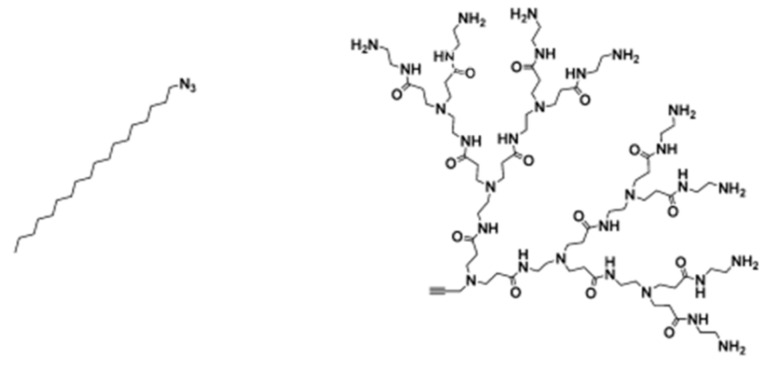
Hydrophobic azide and hydrophilic alkyne click chemistry precursors to Peng-type amphiphilic dendrons. Reprinted with permission from Ref. [112]. Copyright 2012, John Wiley & Sons.

**Figure 17:**
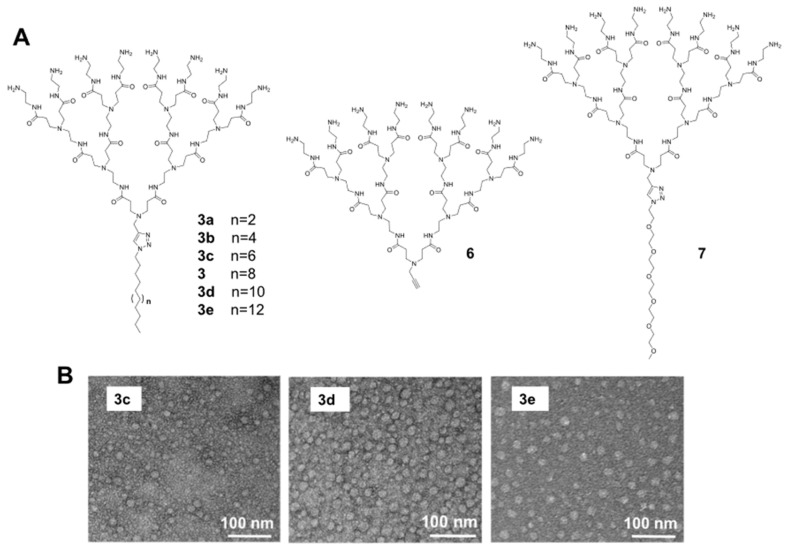
(**A**) Various Peng-type amphiphilic PAMAM dendrons, (**B**) TEMs observed after self-assembly into their respective supramolecular dendrimers, **3c**, **3d,** and **3e** (i.e., nano-micelles). Reprinted and adapted with permission from Ref. [113]. Copyright 2016, John Wiley & Sons.

As early as 2012, L. Peng, et al. [112] developed a groundbreaking and innovative clickable modular component approach for producing self-assembling amphiphilic PAMAM dendrons. This approach involved combining clickable azide and alkyne components. Using these modular components (Figure 16), a wide range of “click-synthesized” amphiphilic PAMAM dendrons may be rapidly synthesized as shown in Figure 17.

These module-clicked amphiphilic Janus PAMAM dendrons could be readily designed and rapidly synthesized according to CNDP engineering principles described in Section 2.3. The desired amphiphilic PAMAM dendrons were readily synthesized in a modular fashion by performing Sharpless-type click coupling of various hydrophilic PAMAM dendrons (i.e., G = 0–3) possessing suitable alkyne focal-point modifications (i.e., hydrophilic-head components) with various azide-modified hydrophobic alkanes (i.e., hydrophobic-tail components) to create large libraries of hydrophilic head-hydrophobic tail and amphiphilic PAMAM dendrons. An abbreviated series of these clickable components with TEMs, as shown in Figure 17.

**Figure 18:**
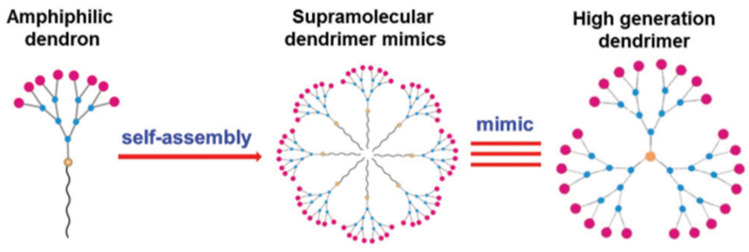
The self-assembly of amphiphilic dendrons into supramolecular dendrimers and proposed mimicry of covalent dendrimers. Reprinted with permission from Ref. [113]. Copyright 2016, John Wiley & Sons.

Chen/Peng et al. have demonstrated that these amphiphilic Janus dendrons readily self-assemble into a wide variety of specific generational-level supramolecular dendrimers (i.e., some refer to them as dendrimeric nano-micelles). These supramolecular dendrimers appeared to mimic the generational-level features and properties of traditional covalently derived dendrimers, G = 4–6, which are generally more onerous and time-consuming to synthesize (Figure 18).

#### 2.5.3. Amphiphilic Dendrimers: Self-Assembly into Dendrimicelles

**Figure 19:**
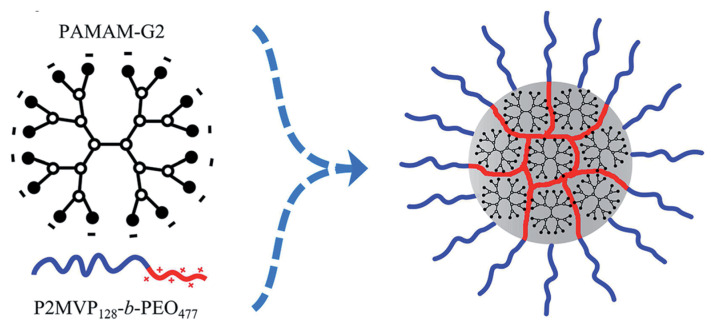
Charge neutralization of a G = 2 anionic surface-charged PAMAM dendrimer with a cationic di-block copolymer to form a poly(dendrimer) micelle. Reprinted with permission from Ref. [115]. Copyright 2014, The Royal Society of Chemistry.

**Figure 20:**
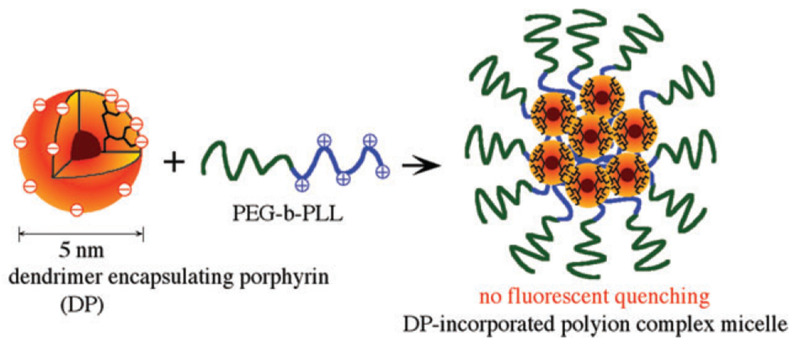
Charge neutralization of an anionic surface-charged PAMAM dendrimer containing an encapsulated porphyrin with a cationic di-block copolymer to form a porphyrin-containing poly(dendrimer) micelle. Reprinted with permission from Ref. [114]. Copyright 2007, The Royal Society of Chemistry.

In an effort to produce modified dendrimer assemblies containing encapsulated photosensitizers (PS) suitable for photodynamic therapy (PDT), certain dendritic entities were prepared by surrounding the porphyrin dye structure with poly(benzyl ether) dendrons possessing peripheral ionic groups [114]. Quite remarkably, these entities spontaneously assembled into poly-ionic complex PIC-type micelles by electrostatic interactions via oppositely charged components of the dendrimer and the ionic block copolymer (Figure 19). Surprisingly, it was also found that these novel dendrimer-encapsulated porphyrin (DP) micelles containing porphyrin dye cores did not self-quench upon irradiation, but instead exhibited amazingly effective in vitro photo-cytotoxicity properties that were found to be very suitable for cancer therapy (Figure 20).

**Figure 21:**
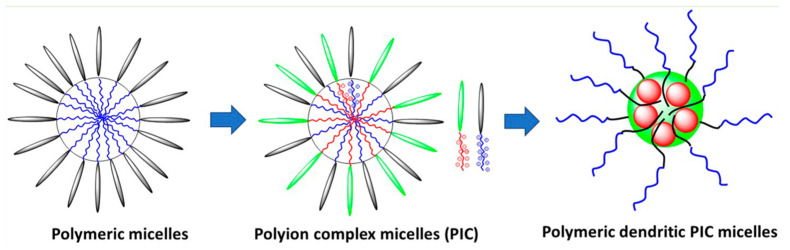
A comparison of polymeric micelles, their conversion into polyion complex micelles (PICs) and their integration into polymeric dendritic PIC micelles. Reprinted with permission from Ref. [116]. Copyright 2021, American Chemical Society.

These dendritic poly-ionic self-assemblies reported earlier by Kataoko were subsequently developed by Velders, et al. as supramolecular PAMAM assemblies and are currently referred to as dendrimicelles. They may be compared with traditional polymeric micelles, polyion complex micelles (PIC), and polymeric dendritic PIC micelles, as shown in Figure 21.

These dendrimicelles are readily formed by first complexing either anionic carboxylic acid-terminated dendrimers with cationic poly(vinylpyridine)-poly(ethylene oxide) di-block copolymers or, alternatively, by combining cationic amine-terminated PAMAM dendrimers with oppositely charged anionic, poly(methacrylic acid)-poly(ethylene oxide) di-block copolymers [115,117]. These amphiphilic dendrimer complexes readily self-assemble into the corresponding dendrimicelles, wherein the aggregation number of the dendrimers in the micellar core of these dendrimicelles may be tuned by varying the dendrimer generation [115] or pH of the environment [118]. Furthermore, it was demonstrated that various metal nanoparticles (i.e., gold, etc.) [117] could be encapsulated within the individual participating PAMAM dendrimers [119,120,121]. This allows the use of transmission electron microscopy (TEM) to monitor the exact localization of the metal nanoparticles. More specifically, it was recently shown that dendrimer-encapsulated gold nanoparticles (AuDENs) could be systematically introduced into these dendrimicelles [117,122,123,124], thus providing a rational strategy for the determination of Au nanoparticle sites and the topologies of these micellar compositions [116,125,126].

**Figure 22:**
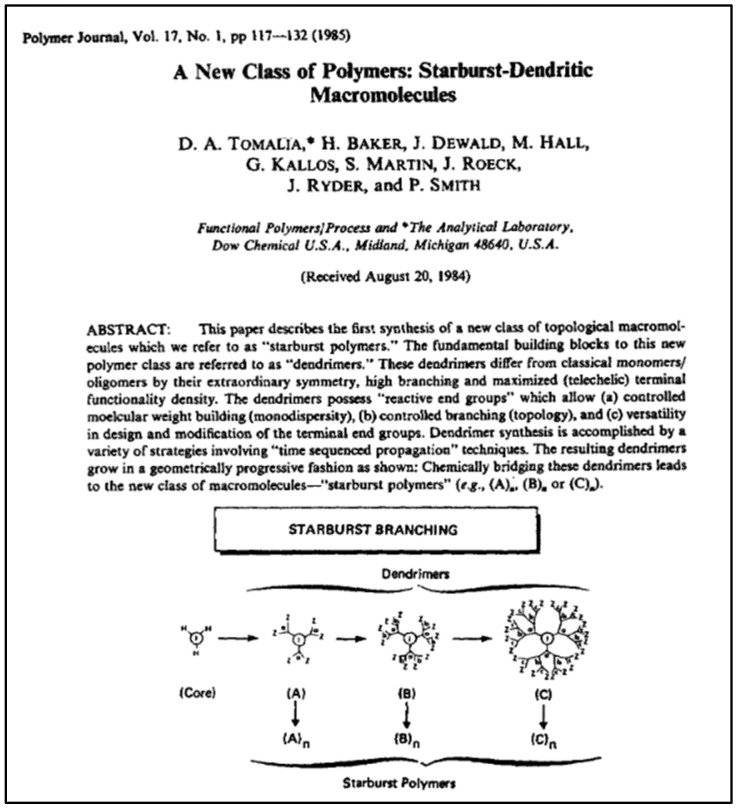
Abstract of original Tomalia, et al. (1985) [31] dendrimer publication describing the initial proposed use of dendrimers as reactive, macromolecular monomers to produce covalent, poly(dendrimer) megamers referred to as “Starburst polymers”. Reprinted with permission from Refs. [15,31]. Copyright 2002, John Wiley & Sons.

Subsequently, Velders, et al. [127] reported the syntheses and isolation of discrete, covalently bridged megameric assemblies reminiscent of the original role we had proposed for dendrimers as discrete and reactive macromolecular monomers from 1979–1984. The original “Starburst Polymer” concept [31] (Figure 22) was to envision dendrimers as macromolecular monomers, which could be covalently linked via their reactive surface groups into a wide range of higher-order macromolecular architectural motifs generally referred to as “Starburst Polymers”. Currently, these poly(dendrimers) or oligo (dendrimers) [27] are referred to as megamers or megameric networks, respectively. Earlier, this concept was clearly demonstrated by the syntheses of core-shell tecto (dendrimers) [45,46], as well as random megamer-type structures, as described later [27].

**Figure 23:**
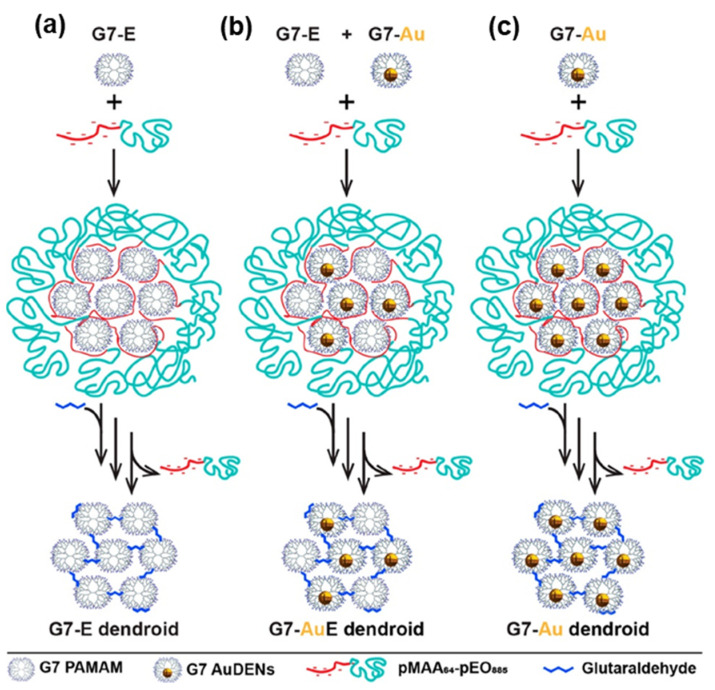
Self-assembly of G = 7 (empty), G = 7 (empty +Au labeled), and G = 7 (all Au labeled) dendrimers into their respective (PIC) supramolecular megamer clusters followed by cross-linking with glutaraldehyde. After removal of the polyionic copolymers, they yielded: (**a**) empty, (**b**) empty-Au-labeled, and (**c**) all Au-labeled covalent, poly(dendrimer), megamers, respectively. Reprinted with permission from Ref. [127]. Copyright 2021, American Chemical Society.

More recently, innovative work by Velder, et al. [127] has advanced this original “starburst polymer concept” to a new level by attaining extraordinary control of poly(dendrimer) complexity via the formation of covalently fixed dendrimicelles. This is accomplished by the discrete labeling of specific dendrimers into Au-loaded and empty forms. This is followed by complexation with polyionic block copolymers, which organizes these selectively functionalized macromolecular, dendrimeric monomers into *dendrimicelle*-type supramolecular assemblies. Subsequent cross-linking of these supramolecular poly(dendrimers) with glutaraldehyde, followed by the release/removal of these poly-ionic block copolymers, yields the respective (a) all-empty, (b) empty-Au-functionalized, and (c) all-Au-functionalized, bridged megameric assemblies, which were each isolated, as illustrated in Figure 23.

These very discretely functionalized megameric assemblies in Figure 23 should probably be referred to as selectively functionalized megamers and not be coined or referred to as dendroids, especially since the term “dendroid” was used to describe special dendritic templates for controlling or amplifying dendritic shapes, as described in earlier work [128].

#### 2.5.4. Dendritic Supramolecular Delivery of Nucleic Acids

Historically, the first covalent dendrimer activity began in the early 1980s, whereas an analogous surge in supramolecular dendrimer development emerged in the late 1980s. The original covalent dendrimer activity was largely initiated and focused in four areas, namely by: (1) the Vögtle, low MWt = 433.6 Da; oligo-PPI dendrimer precursor, cascade structures [22] that inspired the development of enhanced processes for synthesizing macromolecule poly(propyleneimine) (PPI) dendrimers by Meijer, et al. [25] and Mülthaupt [26], (2) the Denkewalter, macromolecular (MW: ~239,000 Da) poly(L-lysine) (PLL) dendrons [129], (3) the Tomalia macromolecular (MWt: ~59,700 Da) poly(amidoamine) (PAMAM) dendrons/dendrimers [31,130], and (4) the Newkome, non-macromolecular, lower-molecular weight (MWt: ~1300 Da) arborols [47].

The extraordinary growth in covalent and supramolecular dendrimer activity during the last decade (~2012–2024) generally parallels a quest for suitable nanoscale delivery vectors for drugs, proteins, and specific nucleic acids beyond DNA (i.e., siRNA, mRNA, etc.). Subsequently, these covalent dendrimer structures were recognized as highly effective cationic nano-vectors for gene delivery by forming charge-neutralized, supramolecular dendriplexes with nucleic acids. That activity began with PAMAM dendrimers (i.e., Szoka [131] and Tomalia/Baker [132,133] (1993–1996)), phosphorous dendrimers (i.e., Majoral/Caminade, 1999) [58,59], PPI dendrimers (Zinselmeyer, 2002) [134], carbosilane dendrimers (Munoz/Bryszewska, 2008) [135], and later with siRNA using PAMAM dendrimers (Peng, 2006) [136].

The first reported dendrimeric supramolecular activity began in the late 1980s with Tomalia/Friberg (1988) [87], as well as Percec [90,137]. It then evolved steadily with a sustained interest in drug-inclusion complexes and DNA delivery, followed by extraordinary growth during this last decade. This recent surge was driven largely by widespread interest in the delivery of smaller single- and double-stranded nucleic acids such as siRNA [138,139] and mRNA [140,141,142]. The siRNA and mRNA structures exhibit distinctly different physico-chemical properties such as size, molecular weight, single-stranded versus double-stranded structures, and conformational flexibility. Although siRNA and mRNA are constructed from the same chemical building blocks and require delivery to the cytoplasm for activity, they are significantly different in size, overall structure (i.e., double- vs. single-stranded), and mechanism of action. That said, an effective siRNA delivery vector may not be suitable for mRNA delivery. The specific targeting and delivery of mRNA is currently an extremely active area of high interest as the result of the recent COVID-19 pandemic, as well as acute interest in the development of advanced vaccines for future pandemic urgencies. These issues are extensively reviewed elsewhere [143]. Beginning in 2016, dendrimer-based mRNA delivery vectors have been at the forefront of recent efforts to develop mRNA vaccines against SARS-CoV [144]. However, liposome-based vaccines developed by BioNTech/Pfizer and Moderna have dominated the COVID pandemic market based largely on their timely development and maturity. These current commercial mRNA delivery vectors require a complex 4-component ionizable lipid system to protect the mRNA from degradation before entering the cell. These commercial 4-component, ionizable lipid-based systems are actually second-generation, self-assembling supramolecular amphiphilic dendrons that appear to have been inspired by the earlier work of Percec et al. [109]. That said, recent breakthrough development by Percec, et al. [145,146] has produced significant advancement to a simplified one-component system based on his sequence-defined ionizable amphiphilic Janus dendrimers (IAJDs). This one-component system has exhibited substantially enhanced efficacy with controlled targeting specificity, room-temperature shelf stability, and a lower cost. The Percec IAJDs co-assemble with mRNA into dendrimersome nanoparticles (DNPs), which may be engineered/programmed to selectively target specific organs, and have been found to be vastly superior to the current four-component, lipid nanoparticle delivery vectors, which are only suitable for mRNA delivery to the liver [143].

By creating a rational library design combined with orthogonal-modular accelerated synthesis and sequence control of their hydrophilic components, Percec has synthesized IAJDs that are performing as extraordinary nano-vectors for the delivery of mRNA to specifically targeted organs. He elucidated the molecular design principles required to target IAJD-assisted mRNA delivery and identified critical functional moieties in the hydrophilic/hydrophobic components of the IAJDs that contribute to the specificity of the targeted organ [147]. This has resulted in ~6-fold enhancements in mRNA delivery levels together with the ability to specifically target key organs such as the spleen, lymph nodes, liver, or lungs [141,146,147,148].

Percec’s discovery of IAJDs represents a significant breakthrough in the development of mRNA therapeutics. His close collaboration with Prof. Drew Weissman (i.e., the 2023 Nobel Prize Laureate for mRNA development) has enabled Percec to innovate and create a large diversity of IAJDs, many of which are described in their brilliant contribution to this current 85th special topic issue [2].

**Figure 24:**
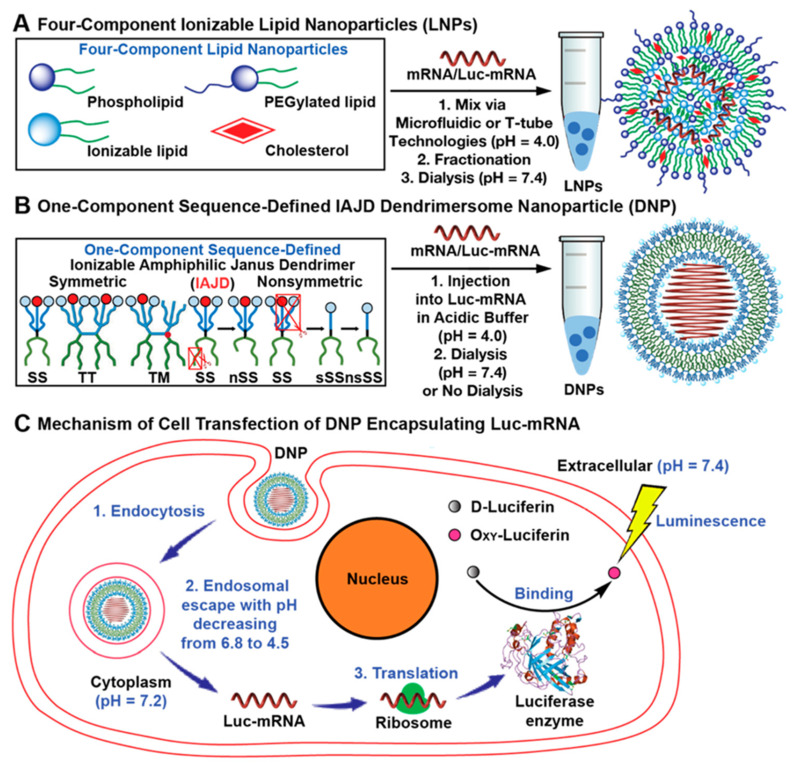
(**A**) A comparison of the current four-component commercial ionizable lipid nanoparticle (LNPs) system with (**B**) the new Percec-type, one-component, sequence-defined IAJD, dendrimersome nanoparticle (DNP) approach. (**C**) A comparison of specific mechanisms involved for the four-component LNP and the one-component DNP nanoparticle approaches, respectively. Reprinted with permission from Ref. [2]. Copyright 2023, MDPI.

Most notable is the development of a simplified one-component, dendrimersome nanoparticle (DNP)-based IAJD system (Figure 24B) compared to the current four-component, lipid nanoparticle (LNP) commercial system, as illustrated below (Figure 24A). An abbreviated cell-transfection mechanism proposed for the (DNP)-based system is described in Figure 24C.

### 2.6. Expected New Application Roles in Pharmaceuticals, Nanomedicine, and Life Sciences

**Figure 25:**
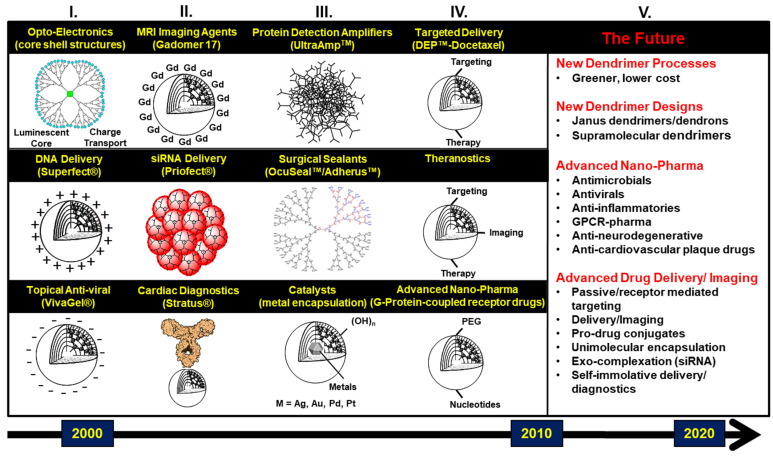
Increased dendrimer structure complexity required for applications defined between 2000–2010) (i.e., Columns (I)–(IV) with predictions concerning future advancements in Column (V) [149].

An overview of DDDS advancements as a function of dendrimer complexity and commercial products (2000–2010) (Columns I-IV), as well as predicted progress during the past decade (2010–2020) (Column V) [28], is as described in Figure 25.

It is both surprising and satisfying to note that many of these 2010 predictions actually occurred, providing a list that includes Janus dendrimers, supramolecular dendrimers, non-traditional intrinsic luminescent (NTIL) dendrimers, dendrimers as specific active pharma ingredients (APIs), and targeted drug delivery, to mention a few.

**Figure 26:**
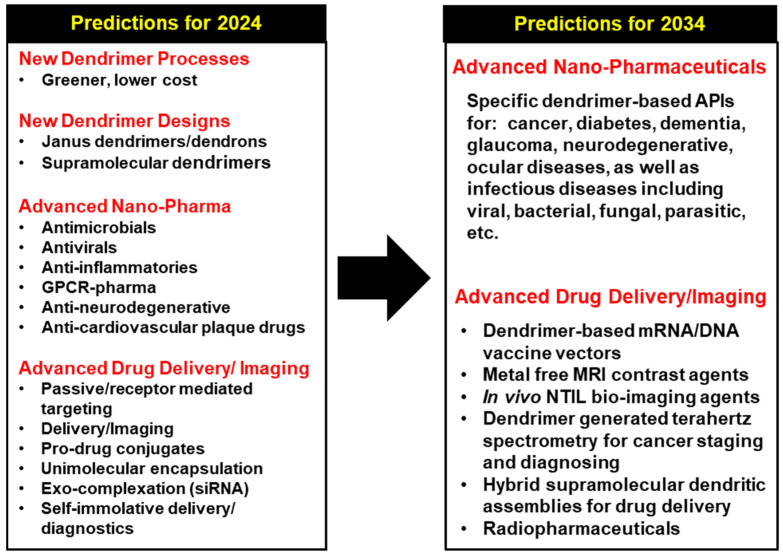
Predicted advancements in pharma, drug delivery, and bio-imaging in 2024 and predicted advancements for 2034.

Based on the current literature and the excellent contributions made to this recent special topics issue, one should expect many more new and exciting application roles for dendrons, dendrimers, and the dendritic state (DDDS) in the next decade [150]. Several conservative predictions for 2024–2034 in pharmaceuticals, nanomedicine, and life sciences are described in Figure 26.

### 2.7. Summary and Conclusions

The ultimate dream and hope for any major scientific discovery as significant as DDDS is that such a scientific breakthrough will evolve into a versatile and beneficial technology platform that will fulfill many unmet societal needs and will enhance the human condition (i.e., health, environmental, etc.). After viewing recent progress in the outstanding contributions to the 85th dendrimer-themed special topic issue, as well as current literature advancements, it provides me with great optimism that this dream and hope for “dendrons, dendrimers, and the dendritic state” will truly be fulfilled.

## Figures and Tables

**Table 1:** Recent trends and topics discussed in contributions to the Pharmaceutics 85th birthday-themed issue on DDDS (2023–2024).

Topics Covered and Discussed	Number of Articles/12
Dendron/dendrimer-CNDPs	12/12
Dendron/dendrimer targeted/drug delivery	8/12
Amphiphilic dendrons/dendrimers, supramolecular assemblies	7/12
Hetero-atom containing dendrimers/dendrons	3/12
Dendritic APIs; anti-(bacterial, viral, fungal, parasite, cancer, neurodegeneration)	3/12
Complement activation	1/12
Dendrimer-inorganic nanoparticle hybrids	1/12

## Supplementary Materials

The following supporting information can be downloaded at: https://www.mdpi.com/article/10.3390/pharmaceutics16121530/s1, Figure S1: Cascade Synthesis/Structure Reproducibility Study.
