# Correction: Tomalia, D.A. Dendrimers, Dendrons, and the Dendritic State: Reflection on the Last Decade with Expected New Roles in Pharma, Medicine, and the Life Sciences. *Pharmaceutics* 2024, *16*, 1530

**DOI:** 10.3390/pharmaceutics17040403

**Published:** 2025-03-24

**Authors:** Donald A. Tomalia

**Affiliations:** 1 The National Dendrimer & Nanotechnology Center, NanoSynthons LLC, Mt. Pleasant, MI 48858, USA; donald.tomalia@gmail.com; Tel.: +1-989-317-3737; 2 Department of Chemistry, University of Pennsylvania, Philadelphia, PA 19104, USA; 3 Department of Physics, Virginia Commonwealth University, Richmond, VA 23284, USA

## 1. Missing Citation in the Main text

In the original publication [1], there were mistakes related to reference citation and information. Citations [28,63,72,73], [107], and [75] were not cited. They have now been inserted in the captions of Figures 7 and 11 and in Section 2.4, paragraph 5.

## 2. Incorrect Citation in the Main Text

The following citation errors were identified and have now been corrected as follows:

- Section 2.5.1, Paragraph 1: Citations [99–101] and [100] were incorrectly cited. The correct citations are now [99–103] with two citations added, and [102].
- Section 2.5.1, Paragraph 2: Citation [100] was incorrect. It has now been corrected to [99] Section 2.5.1, Paragraph 3: Citation [17,104,105] has been updated to [17,104–106], with one citation added.
- Section 2.5.1, Paragraph 4: Citation [100] has been corrected to [99], while citation [107,108] has been updated to [107–109] with one citation added.
- Section 2.5.3, Paragraph 4: Citation [49] has been changed to [27].
- Section 2.5.4, Paragraphs 1–4: Citation [130] has been updated to [31,130] with one citation added, while citation [58] has been changed to [58,59] with one citation added, citation [87,137] has been corrected to [87] with one citation removed, and citation [90,138] has been corrected to [90,137].

## 3. References Correction

In the original publication [1], the reference information for [28] and [150] was incorrect. These references have now been corrected as follows:

28. Tomalia, D.A.; Christensen, J.B.; Boas, U. *Dendrimers, Dendrons, and Dendritic Polymers: Discovery, Applications and the Future*; Cambridge University Press: New York, NY, USA, 2012; pp. 1–412.
150. Singh, M.K. Dendrimer Technology: Concept to Cutting-Edge Applications, 57th SPSR International Webinar on Dendrimer Technology. Available online: https://www.youtube.com/live/sAUfDi8XI64?app=desktop&feature=share (accessed on 21 July 2024).

Additionally, the original publication was missing references [50] and [137], which have now been included:

50. Aharoni, S.M.; Crosby, C.R., III; Walsh, E.K. Size and Solution Properties of Globular tert-Butyloxycarbonyl-Poly(α,iε-L-Lysine). *Macromolecules* **1982**, *15*, 1093–1098.
137. Hudson, S.D.; Jung, H.-T.; Percec, V.; Cho, W.-D.; Johansson, G.; Ungar, G.; Balagurusamy, V.S.K. Direct Visualization of Individual Cylindrical and Spherical Supramolecular Dendrimers. *Science* **1997**, *278*, 449–452.

Furthermore, there was a duplication related to references [95] and [96], as reference [96] was reprinted from reference [95]. As a result, reference [95] has been removed from the list.

With this correction, the order of some references has been adjusted accordingly. The authors state that the scientific conclusions are unaffected. This correction was approved by the Academic Editor. The original publication has also been updated.
