# Supplementary material for: Dendrimers, Dendrons, and the Dendritic State: Reflection on the Last Decade with Expected New Roles in Pharma, Medicine, and the Life Sciences
Source: Pharmaceutics. 2024 Nov 28;16(12):1530. doi: 10.3390/pharmaceutics16121530 (PMC11676903; doi:10.3390/pharmaceutics16121530)
Supplement: Supplementary file 1 [file pharmaceutics-16-01530-s001.zip › pharmaceutics-3306854-supplementary.pdf]

Figure S1: Cascade Synthesis/Structure Reproducibility Study

DECLARATION

STATE OF MICHIGAN)
County of Midland) ss.
U.S.A.)

I, Dr. Lars Piehler, declare and state:

THAT, Dr. D. A. Tomalia, an inventor of the relevant Japanese patent application assigned to the Dow Chemical Company, requested that I perform the experimental works described herein;

THAT, the results of the experimental work would be submitted to the Japanese Patent Office relevant to the STARBURST™ technology;

THAT, the purpose of the experimental work was to reproduce the synthesis of branched polyamines described by Moors and Vögtle (Moors, R.; Vögtle, F. *Chem. Ber.* **126**, 2133 (1993), copy attached). The authors report a method to reduce intermediate polynitrile compounds to polyamines that is superior to the earlier published method by Vögtle et al. (Buhleier, E.; Vögtle, F. et al. *Synthesis* **155** (1978), copy attached). I used the new reduction method to synthesize branched polyamines so that they could be compared to the branched polyamine products synthesized by Christoph Rickert at the Michigan Molecular Institute (MMI) by the older method.

THAT, the following experimental discussion and results have been performed by me and discussed with Dr. Tomalia.

DECLARATION

STATE OF MICHIGAN)
County of Midland) ss.
U.S.A.)

I, Dr. Christoph Rickert, declare and state:

THAT, Dr. D.A. Tomalia, an inventor of the relevant Japanese patent application assigned to The Dow Chemical Company, requested that I perform the experimental works described herein;

THAT Dr. Tomalia informed me that the results of the experimental work would be submitted to the Japanese Patent Office relevant to the STARBURST ™ technology;

THAT the purpose of the experimental work was to reproduce the synthesis of the branched polyamines (so called cascade molecules) described by Vögtle et al. (Buhleier, E.; Vögtle, F. et al., *Synthesis* 155 (1978), copy attached) In the course of the development of new branched polyaza compounds as multidentate ligands for host-guest applications, the authors described a procedure to build up these molecules in a stepwise manner. I tried to reproduce these results and this Declaration contains my data from that effort;

THAT, I chose benzylamine (**1**) for the core molecule as described by Vögtle (*supra*), because it represents the most simple case (It is a mono amine and therefore the molecule just grows in one direction at a time.);

THAT the following experimental discussion and results have been performed by me and discussed with Dr. Tomalia.

Experimental Data

Measurements:

Nuclear magnetic resonance (NMR): Varian Unity 300; All spectra were recorded with chloroform-d as solvent. Tetramethylsilane was used as internal standard for ^{1}H-NMR (δ=0 ppm). The chloroform signal was set to δ=77 ppm for ^{13}C-NMR.

Mass spectroscopy (MS): Finnigan TSQ 700 with electrospray inlet; conditions: 1.5 μL/min, methanol/water 95:5

Infrared spectroscopy (IR): Nicolet FT IR 20 DXB; Spectra were recorded as films on KBr discs.

Thin layer chromatography (TLC): Whatman flexible silica gel plates were used (PE SIL G/UV, polyester support). They were stained with iodine.

Chemicals:

Benzylamine (Aldrich, 99%; 18,570-1); acrylonitrile (Aldrich, 99+%; 11,021-3; Lot 05808HZ), methanol (Fisher, certified ACS; A412-20; Lot 933472), cobalt(II)chloride hexahydrate (Aldrich, 98%; 19,807-2; Lot 06513 KF), conc. HCl solution (Fisher; A142-212; Lot FL030589), aluminum oxide (Aldrich, activated, neutral, Bockman I; 19,997-4; Lot 07004 PZ), silica gel (Aldrich, 200-400 mesh, 60 Å; 28,859-4; Lot 14421AF), acetic acid (Aldrich, 98%; 10,908-8; Lot 00419 EF), chloroform (Fisher, HPLC grade, pentene stabilized; C607-4; Lot 920604), ammonium hydroxide (Fisher reagent ACS; A669-212; Lot 935232), magnesium sulfate (Fisher, certified, anhydrous; M65-500; Lot 930247B), sodium sulfate (Fisher,certified ACS, anhydrous; S421-500; Lot 886915)

The various compound numbers refered to below can be found in the reaction Schemes.

*Synthesis of bis-nitrile **(2)**: (Method b))*

A solution of 30 ml (0.46 mol) acrylonitrile in 20 ml methanol were added to a solution of 15.0121 g (0.140091 mol) freshly distilled benzylamine (**1**) in 50 ml methanol. The solution was first stirred for 26 h at 42 °C under a nitrogen atmosphere. TLC on silica gel indicated (CH_2Cl_2/ethyl acetate 4:1) that there was still some mono-adduct present. Another 10 ml (0.15 mol) acrylonitrile were added to the reaction mixture and the resulting mixture was heated to

75 °C for 3 1/2 days. At this time 2.2% mono-adduct was found by ^{1}H-NMR. After removing the solvents, 34.2298 g of a slightly yellow oil were isolated. Now, 10.2946 g of this crude product was chromatographed on silica gel with CH_2Cl_2/ethyl acetate, 8:1. This gave 8.8269 g of an slightly yellow oil. In addition to the peaks for the desired product, peaks for the Michael addition product of methanol to acrylonitrile were found in the NMR spectra. This material was dissolved in ethyl acetate and extracted twice with 1N HCl solution. The acidic layer was made basic with NaOH solution, followed by extraction with ethyl acetate (2x). The organic layer was washed with brine and dried with magnesium sulfate. Removing of the solvent gave 6.5760 g of bis-nitrile (**2**) as a colorless oil that was >98% pure as analyzed by H-NMR spectroscopy.

^{1}H-NMR (300 MHz): 7.40-7.20 (m, 5H), 3.68 (s, 2H), 2.85 (t, J=6.7 Hz, 4H), 2.41 (t, J=6.7 Hz, 4H)

^{13}C-NMR(75 MHz): 137.48 (s), 128.32 (d), 128.31 (d), 127.35 (d), 118.39 (s), 57.79 (t), 49.13 (d), 16.44 (d)

*Synthesis of bis-amine (**3**):*

Trial 1: To a solution of the 1.0603 (4.971 mmol) bis-nitrile (**2**) and 4.7628 g (20.018 mmol) cobalt(II)chloride hexahydrate in 40 ml methanol, 7.5614 g (199.88 mmol) sodium borohydride was added in small portions during 1 h. The reaction mixture started boiling and violent gas evolution with the deposition of a black solid was observed. After the addition of about 2/3 of the sodium borohydride, the mixture was too viscous to be stirred. Therefore another 20 ml of methanol were added. After the addition was completed the very viscous mixture was stirred for 2 h, then 40 ml conc. HCl solution were added slowly. Solvents were removed from the blue-green mixture followed by the sequential addition of 50 ml conc. ammonium hydroxide solution and 50 ml chloroform. At this point an insoluble precipitate was filtered off. The precipitate was washed with another 50 ml chloroform and the filtrate extracted four times with a total of 100 ml chloroform. The organic layer was dried with magnesium sulfate. Removing of the solvent gave 0.5224 g (47 %) of a brown oil. Analysis of this material by MS, ^{1}H and ^{13}C NMR proved it to be a mixture of several different products including the expected amine (**3**).

Trial 2: To a solution of the 4.5783 (21.463 mmol) bis-nitrile (**2**) and 20.4532 g (85.963 mmol) cobalt(II)chloride hexahydrate in 170 ml methanol, 32.4761 g (858.48 mmol) sodium borohydride was added in small portions during 1 h. Violent gas evolution and the deposition of a black solid was observed. The reaction mixture was cooled with an ice bath in order to prevent methanol from evaporating. After the addition of about 2/3 of the sodium borohydride, the mixture was too viscous to be stirred. Therefore another 40 ml of methanol were added. After the addition was completed the very viscous mixture was slowly agitated with the Rotavap for 2.5 h, then 150 ml conc. HCl solution were added slowly. Solvents were removed from the blue-green mixture (Rotavap, 34 °C) then 200 ml conc. ammonium hydroxide solution were added and insoluble precipitate filtered off. The precipitate was washed with 15 ml chloroform and the filtrate extracted six times with a total of 400 ml chloroform. The organic layer was dried with magnesium sulfate. Removing of the solvent gave 3.5696 g (75 %) of a brown oil. Analysis of this material by MS, ^{1}H and ^{13}C NMR proved it to be a mixture of several different products including the expected amine (**3**).

*Synthesis of tetra-nitrile (**4**): (Method a))*

Acetic acid (3.1374 g; 52.246 mmol) was added to a solution of the amine (**3**) (2.8914 g; 13.060 mmol) in 65 ml acrylonitrile. The solution was heated to reflux for 24 h under nitrogen atmosphere. Some insoluble material was formed during that time. After removing the solvents, the oil was dissolved in 50 ml chloroform and extracted with 10 ml conc. ammonium hydroxide, washed with water and dried over sodium sulfate to give a crude yield of 4.7729 g (84.3 %) of slightly brown oil.

4.5082 g of this oil were chromatographed on 200 g aluminum oxide with chloroform. Fractions of 75 ml were collected. The following fractions were unified: **A**: Fractions 5-13: 60.3 mg; **B**: Fractions 14-25: 86.0 mg; **C**: Fractions 26-40: 39.9 mg; **D**: Fractions 41-64: discarded (TLC indicated that they contained even much less material than fractions 26-40)
This gave a total of only 186.2 mg after passing 4.8 L chloroform through the column. Since most of the material was still on the column, a methylene chloride/methanol 20:1 was chosen as eluent. This gave the following samples: **E**: Fraction 65: 0.1691 g; **F**: Fraction 66: 2.2459 g; **G**: Fraction 67: 1.0506 g; **H**: Fractions 68-76: 0.2601 g

All fractions together were 3.9119 g which corresponds with 86.8% of recovered material. **A** - **H** were characterized each by electrospray MS, ^1H and ^{13}C-NMR spectroscopy.

*Synthesis of tetra-amine (**5**):*

A sample of tetra-nitrile (**4**), independently prepared by L. Piehler at Michigan Molecular Institute (MMI), was purified by an improved procedure on aluminum oxide (chloroform/ethyl acetate 4:1, then changed to chloroform/ethyl acetate 1:1) followed by a second chromatography on silica gel (methylene chloride/methanol 40:1). This gave a fairly pure sample as could be shown by electrospray MS, ^1H and ^{13}C-NMR.

Sodium borohydride (3.0281 g; 80.045 mmol) was added in small portions to a solution of 0.4269 g (0.984 mmol) of the purified tetra-nitrile (**4**) and 1.8753 g (7.882 mmol) cobalt(II)chloride hexahydrate in 8 ml methanol. Since the reaction mixture turned into a solid after addition of about one third of the hydride, more methanol had to be added in order to be able to mix the hydride with the black precipitate. Therefore two more portions of 8 ml methanol were added. After completion of the addition, the mixture was stirred for 1 h, then 8 ml conc. HCl solution were added. After removing the solvents, 10 ml conc. ammonium hydroxide solution was added. Insoluble salts were filtered off and washed with chloroform. The ammonium hydroxide solution which turned dark-brown very rapidly during the filtration, most likely due to oxidation of Co(II) to Co(III) by air, was extracted six times with a total of 200 ml chloroform. Drying of the organic layer with magnesium sulfate and removing of the solvent gave 0.1062 g (24.0 %) of a yellow oil. The product was characterized by electrospray MS, ^1H and ^{13}C-NMR.

Discussion and Results

The conversion of benzylamine (**1**) to the bis-nitrile (**2**) proceeded without difficulties. Two different methods were used. (Scheme 1) Method a), using acetic acid and acrylonitrile as solvent, gave complete conversion in 24 h. (Method a) was investigated by L. Piehler in our Lab at MMI.) Method b) was based on reaction conditions similar to the one used for the synthesis of STARBURST ™ PAMAM dendrimers. Acrylonitrile was used with methanol as

solvent. Method b) was much slower but gave the same product as can be shown by ^{1}H and ^{13}C-NMR. Purification, i.e. removal of a small amount of mono-adduct, was easily achieved by chromatography.

Reduction of the bis-nitrile (2) was done using Vögtle's exact condition (*supra*). During addition of the sodium borohydride to the solution of Co(II)chloride and the nitrile (2) in methanol, a black precipitate was formed. This precipitate was examined by Heinzman et al. in *J. Am. Chem. Soc.* 104 (1982), at 6801 and by Osby et al. in *J. Am. Chem. Soc.* 108 (1986), at 647. They found that cobalt borate (Co_2B) precipitates from methanol solution. The reduction of the nitrile then takes place on the surface of the Co_2B in the presence of sodium borohydride. The major problem encountered following Vögtle's procedure (*supra*) was to get the hydride in solution since the reaction mixture was so viscous that the solid remained on the surface and stirring was not possible. Addition of more methanol was a necessity.

Scheme 1: Synthesis of tetra-amine (5)

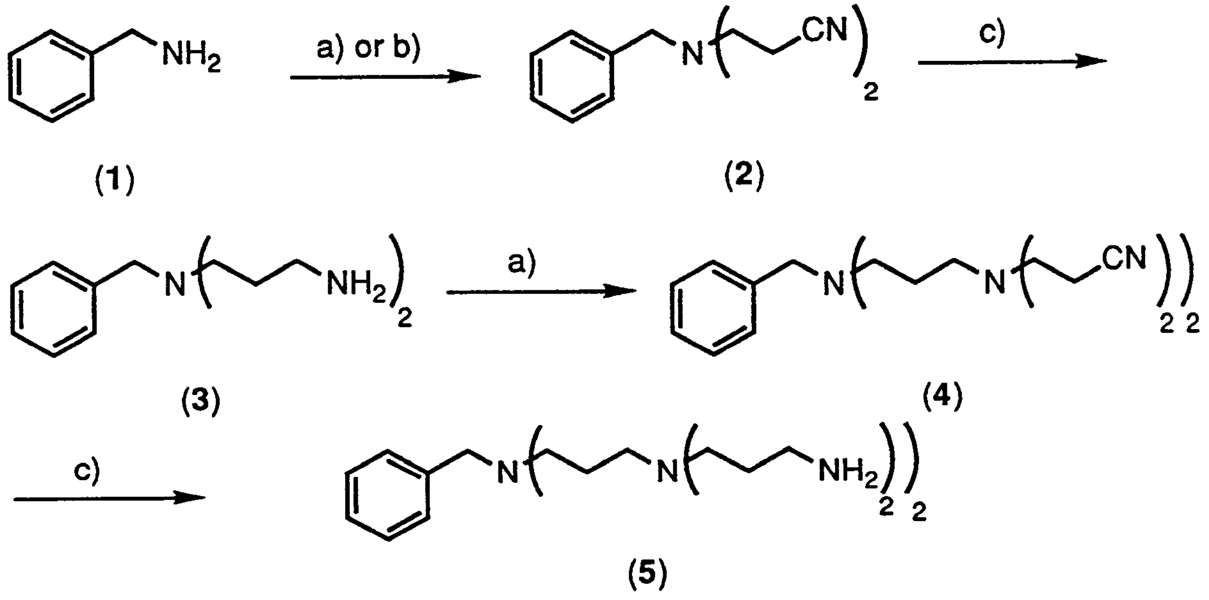

a) acrylonitrile/acetic acid, reflux, 24h

b) methanol/acrylonitrile, 75 °C, 3.5 days

c) Co(II)/$NaBH_4$/methanol

The reduction (step c)) of bis-nitrile (2) gave a mixture of different products as easily can be seen from the electrospray mass spectroscopy (MS) shown in Fig.1.

Fig. 1: Electrospray MS of crude product of the reduction of nitrile **(2)**

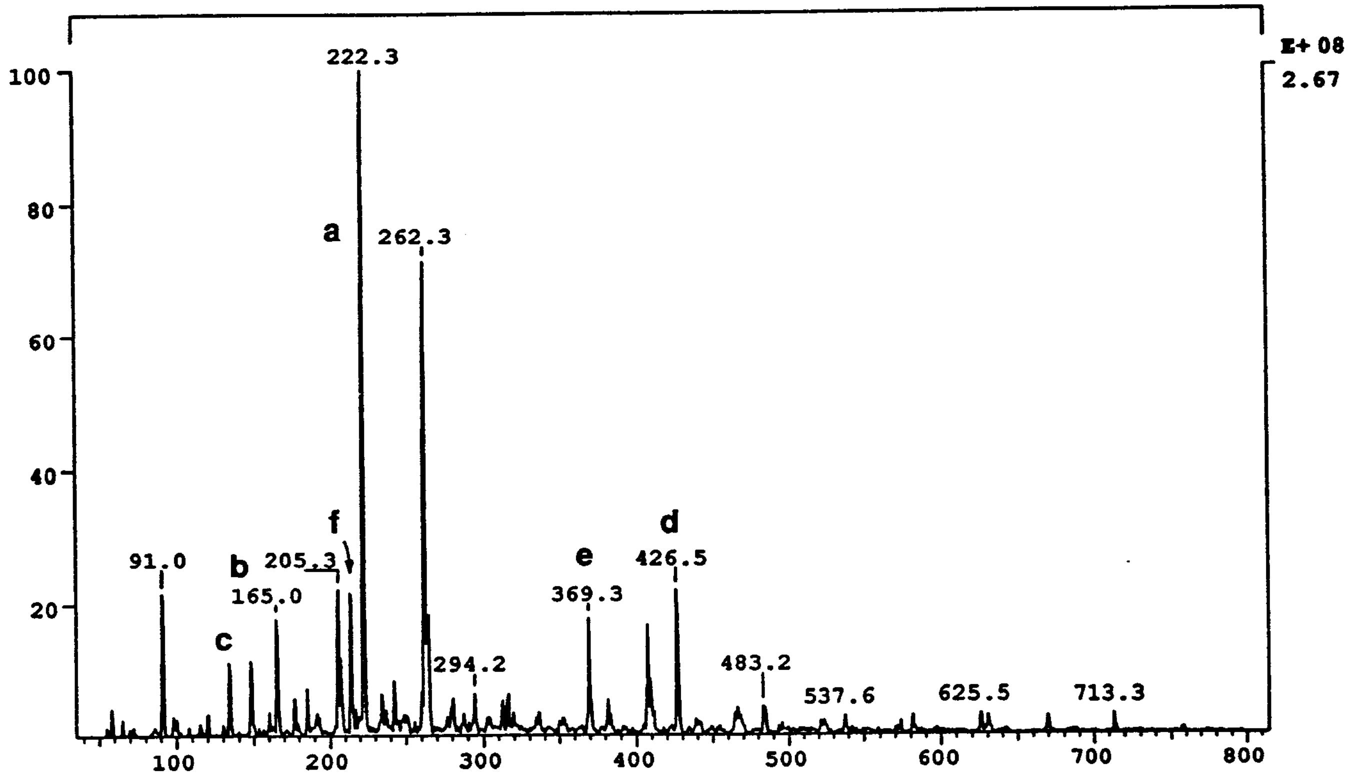

a:

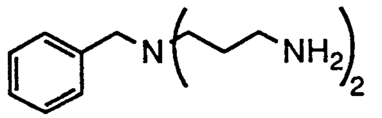

$C_{13}H_{25}N_3$ (221.39)

b:

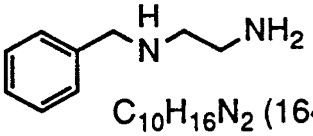

$C_{10}H_{16}N_2$ (164.28)

c:

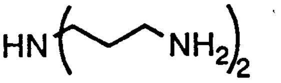

$C_6H_{17}N_3$ (131.14)

d:

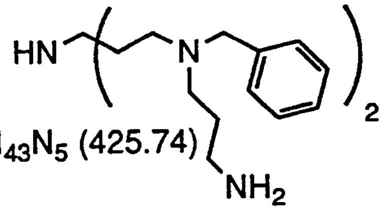

$C_{20}H_{43}N_5$ (425.74)

e:

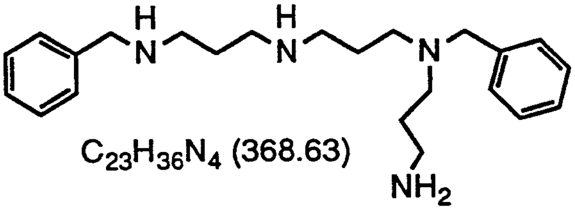

$C_{23}H_{36}N_4$ (368.63)

f:

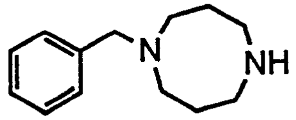

$C_{13}H_{27}N_2$ (211.42)

The main product is the bis-amine (**3**) (MW=221). (Under the conditions used in the MS experiment, the amines pick up a proton from the aqueous solution. Therefore the peaks found in the spectra are always at (M+1)/z. An exception are the amines complexes with alkali metals (Na^+, K^+) that provide the charge. In these cases it is either (M+23)/z or (M+39)/z).) Several peaks for defect structures were found. Cleavage of one or more arms was observed as well as dimer formation and cyclisation. The reduction was complete as indicated by the complete absence of a resonance at 2248 cm^{-1} for the cyano group in the infra red (IR) spectra.

Following the literature procedure (Buhleier, E.; Vögtle, F. et al., *Synthesis* 155 (1978)), the crude product of the reduction was used for the alkylation with acrylonitrile. This reaction caused no problems. Since the bis-amine (**3**) was not purified, a mixture of different nitriles was isolated. Following Vögtle's procedure (*supra*), they were chromatographed on neutral aluminum oxide with chloroform as eluent. No activity of the aluminum oxide used was reported. Sixty-four fractions were taken which corresponds to 4.8 L of chloroform passed through the aluminum oxide to give four samples (**A**-**D**). The total of these fractions contained only 4.1% of the initial material put on the aluminum oxide. All were analyzed by electrospray MS, ^{1}H and ^{13}C NMR. (Fig. 2-4)

Fig. 2a: Electrospray MS of samples **A-C**

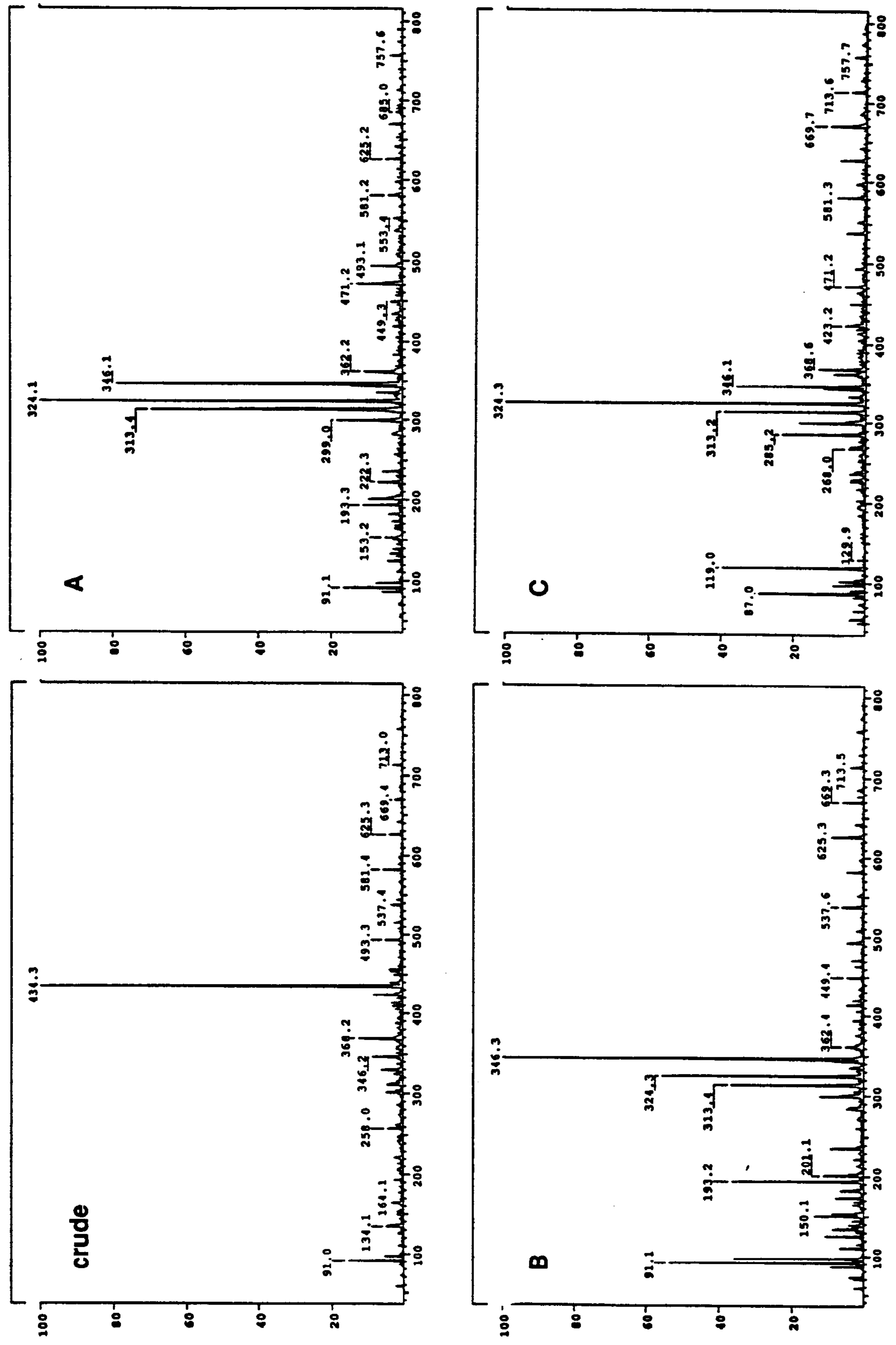

Fig. 2b: Electrospray MS of samples **E**-**H**

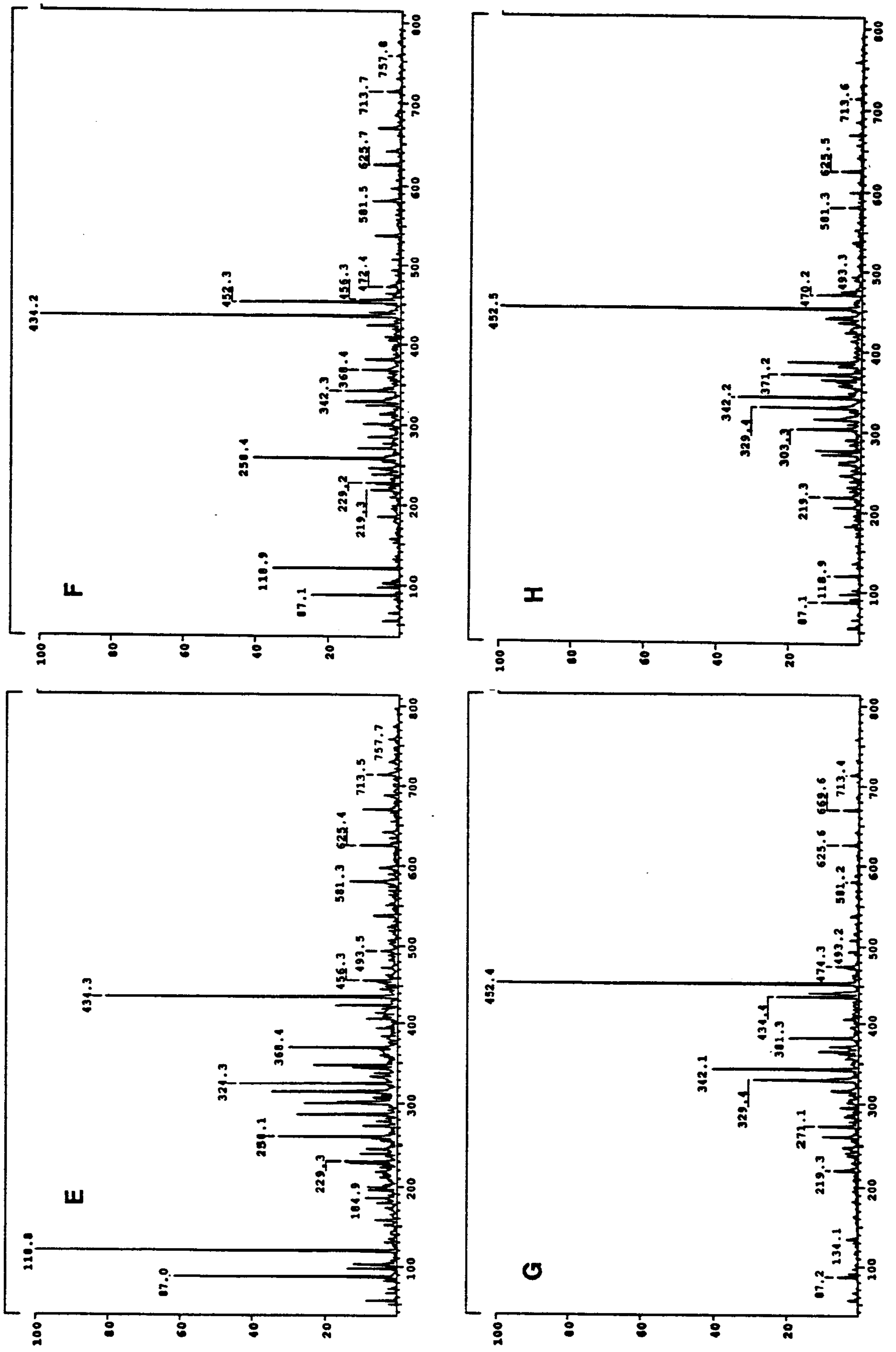

Fig. 3: ^{1}H NMR of samples **A-H**

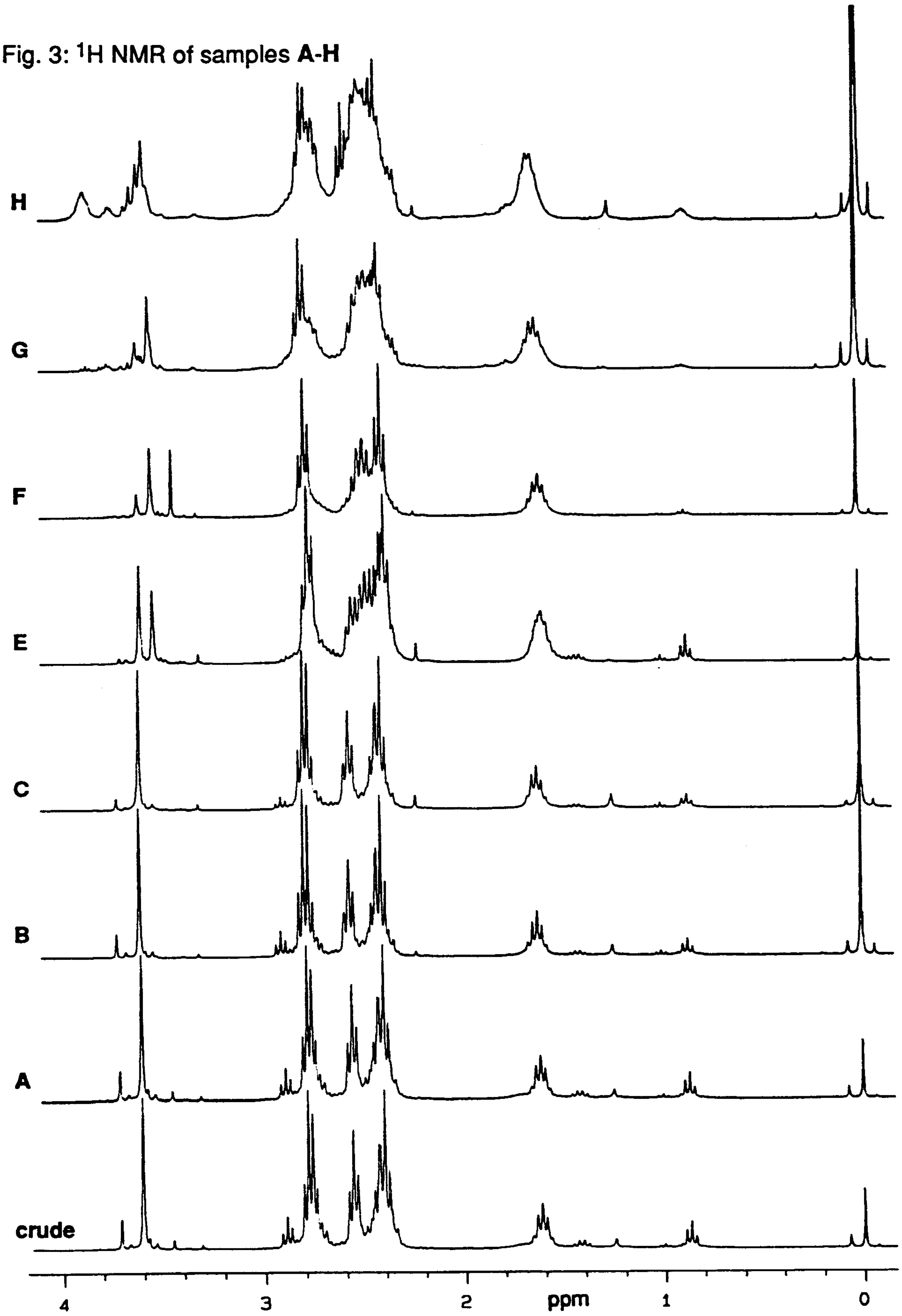

Fig. 4: ^{13}C NMR of samples **A-H**

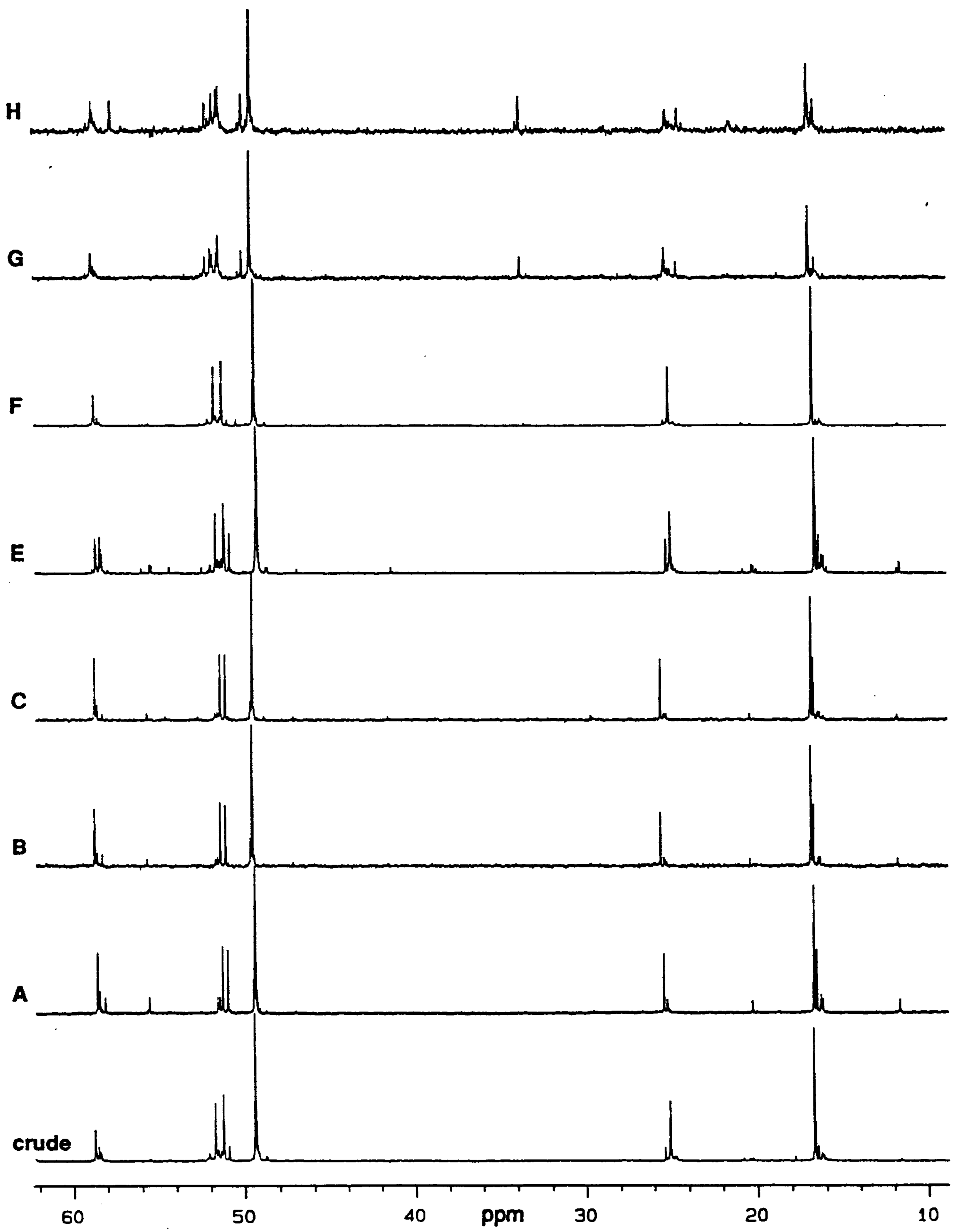

Conclusions

THAT, all analytical methods proved that these samples were very impure. In the MS traces, no peak for the tetra-nitrile (**4**) was found, indicating the complete absence of the desired product. This means that it was not possible to isolate any of the nitrile (**4**) by following this procedure. Therefore no tetra-nitrile (**4**) was obtained following Vögtle's literature procedure (*supra*).

THAT, after the complete failure of Vögtle's procedure (*supra*), we devised a solvent mixture that allowed the removal of the residual material still sticking on the aluminum oxide. By doing so it was possible to recover 86.5% by weight of a complex product mixture. (Samples **E-G** in Fig. 2-4) None of the fractions was pure. All fractions consisted of a wide variety of different products, among them the nitrile (**4**) in different amounts.

THAT, since none of these fractions was suitable for the subsequent reduction, an authentic sample of (**4**) of high purity was prepared by L. Piehler at MMI by a modified approach. The reduction of nitrile (**2**) was done the same way but completely different conditions were applied to the chromatography of the crude nitrile (**4**). First it was purified using aluminum oxide with chloroform/ethyl acetate as eluent followed by a second purification using silica gel with methylene chloride/methanol. In this way a fairly pure sample was received. (Fig. 5 a, below)

THAT, reduction of this nitrile gave a broad mixture of products as can be seen from the electrospray MS. (Fig. 5 b) Note that none of the tetra-amine (**5**) was present in this mixture. No signal at (m+1)/z=450 was found. (Fig. 5 b, below) Identical side reactions as in the first reduction step took place in this step. But this time they were by far dominating. Therefore even starting from pure nitrile (**4**) this reduction did not yield any tetra-amine (**5**). The major peak found in the MS represents a cyclization product. One would expect that the cyclization process would dominate as shown in Scheme 2 since as more amines are present in close proximity on the same molecule, thus excluding the formation of "cascade type branching".

Fig. 5: Electrospray MS of pure tetra-nitrile (**4**) and of the crude reduction product

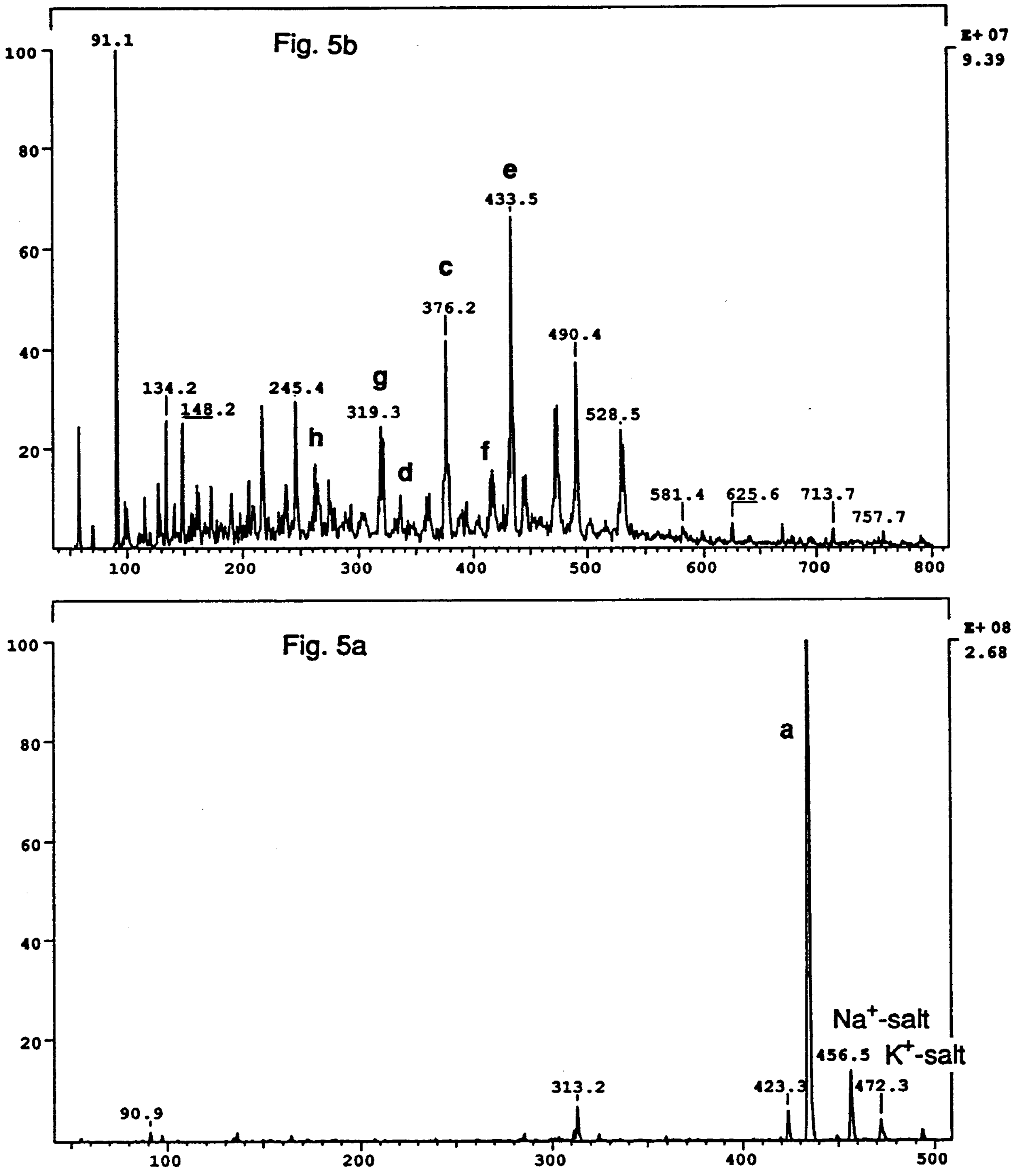

5a:

a: 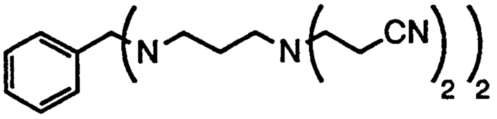

$C_{25}H_{35}N_7$ (433.67)

Na^+-salt (456.30)

K^+-salt (472.40)

5b:

b: 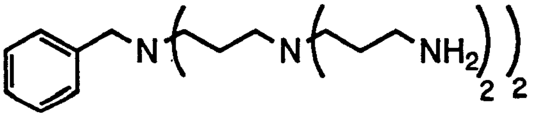

$C_{25}H_{51}N_7$ (449.54)

c: 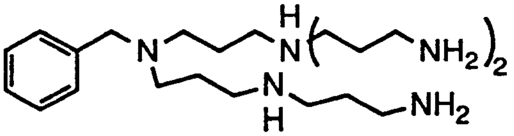

$C_{22}H_{41}N_5$ (375.68)

d: 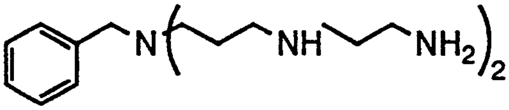

$C_{19}H_{37}N_5$ (335.61)

e: 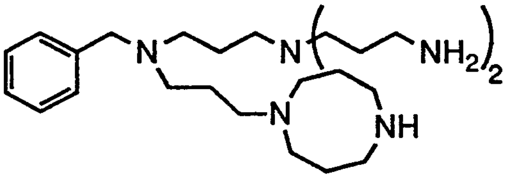

$C_{25}H_{48}N_6$ (432.79)

f: 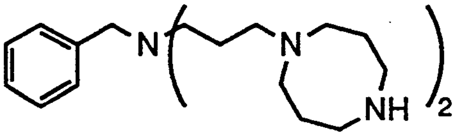

$C_{25}H_{45}N_5$ (415.75)

g: 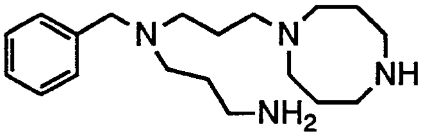

$C_{19}H_{34}N_4$ (318.57)

h: 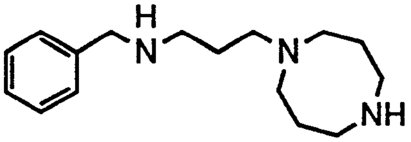

$C_{16}H_{27}N_3$ (261.46)

Scheme 2: Proposed mechanism for the intra-molecular cyclisation during the reduction of nitriles (**2**) or (**4**)

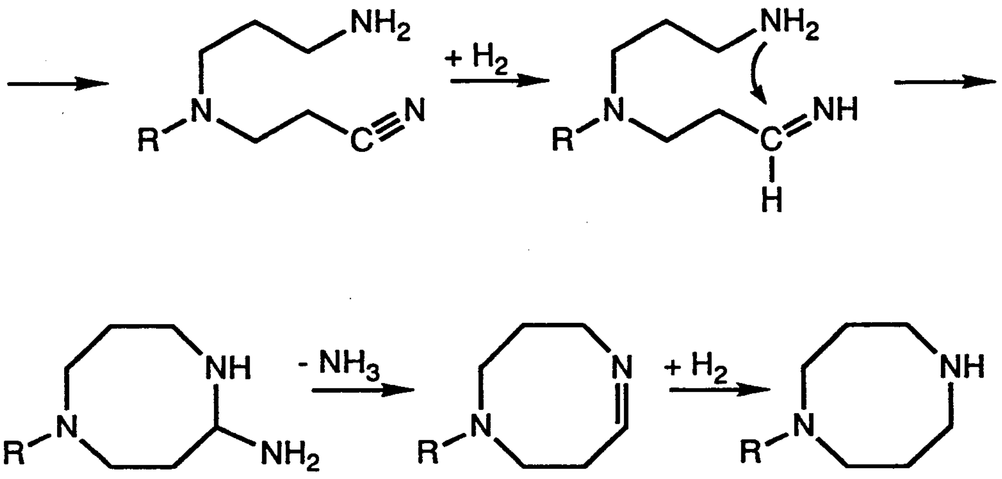

THAT, therefore in no instance could the results of Vögtle (*supra*) be reproduced. The tetra-amine (**5**) could not be detected even at parts per million by use of highly sensitive electrospray MS techniques. For that reason, the compounds now claimed by the present Japanese patent application could not previously have been known or prepared by the process described in the Vögtle article published in 1978 (*supra*).

The undersigned DECLARANT, Christoph Rickert, declares further that all statements made herein of his own knowledge are true and that all statements made on information and belief are believed to be true; and further that these statements were made with the knowledge that willful false statements and the like so made are punishable by fine or imprisonment, or both, under Section 1001 of Title 18 of the United States Code.

Date: 12-9-93 Christoph Rickert

Christoph Rickert, Ph. D.

EXPERIMENTAL DATA

General Synthetic Chemistry: All reactions were performed under a dry nitrogen atmosphere. Oxygen and moisture-sensitive liquids and solutions were transferred to reaction flasks by syringe or canula through rubber septa. Unless otherwise noted, reactions and manipulations were carried out at room temperature. Concentration of solutions was accomplished using a Büchi rotary evaporator under vacuum, usually followed by a final removal of residual solvents at 0.1 mm Hg on a vacuum line.

Reagents and Solvents: Benzylamine (Aldrich, 99%), was distilled before use; all other chemicals were used without further purification. The following were obtained from Fisher Scientific: Methanol (certified ACS), conc. ammonia solution (reagent ACS), chloroform (HPLC grade, pentene stabilized), sodium sulfate (certified ACS), methylene chloride (certified ACS). The following were also obtained from Aldrich: acrylonitrile (99+ %), cobalt chloride hexahydrate (98%), alumina (activated, neutral, Brockman I), acetic acid (98%), acetonitrile (99%), diisobutylaluminum hydride (1.5 M solution in toluene).

Spectroscopic Measurements: Proton (^{1}H NMR) and carbon-13 (^{13}C NMR) nuclear magnetic resonance spectra were recorded on a Varian Unity 300, using deuterated chloroform (Aldrich) as solvent. Chemical shifts were reported (in parts per million) relative to internal tetramethylsilane (δ=0.0 ppm) for ^{1}H NMR and to $CDCl_3$ (δ=77.0 ppm) for ^{13}C NMR. Mass spectra (MS) were recorded on a Finigan TSQ 700 with electrospray inlet; conditions: 1.5 μl/min, methanol/water 95:5 .

(It is important to mention at this point that electrospray mass spectroscopy is a very powerful and reliable way to detect the presence of large, charged molecules. It can be used to detect organic compounds such as amines at levels less than 10 ppb in complex matrices (see Goldner, H. J. *R & D Magazine* , Feb. 1993, p 42; copy attached). The following short article (Schilling, A. *R & D Magazine*, Feb. 1993, p. 44) describes the basic principles of this analytical technique:)

The Fundamental Principles of Electrospray Operation

Electrospray mass spectrometry (ESMS) is a soft ionization technique that allows the analysis of large biological molecules as well as smaller polar compounds.

In a typical ESMS instrument, a solvent flow of a few µl/min is introduced into an ion source chamber held at atmospheric pressure. The solvent flow forms a spray of fine droplets in response to a large applied electric field.

As these droplets evaporate, analyte ions whose polarity is opposite to the applied potential migrate to the surface of the droplets, where Coulomb repulsion causes the droplets to break up into yet smaller droplets, greatly enhancing evaporation.

At some minimum droplet diameter, analyte ions are believed to desorb from the droplets into the gas phase. This process is collectively known as ion evaporation, and is the primary mechanism for gas-phase ion formation in electrospray.

The key to whether a compound of interest will yield an observable ion depends on its ability to ionize in solution. Thus, basic compounds are dissolved in acidic solutions and acidic compounds in basic solutions. The resulting cations and anions are analyzed in positive and negative ion modes, respectively.

Neutral compounds also can be ionized by forming adducts with cations or anions that are added to the solution as salts.

To change from one mode to another requires only a change in the polarity of the potential. Compounds that work best are generally polar and water-soluble to some extent.

Quadrupole mass spectrometers typically have upper mass limits of about 4,000 daltons. These instruments separate ions on the basis of their mass-to-charge ratios.

Most large biological molecules such as proteins and DNA are polyelectrolytes, capable of carrying many charges in solution. As a result, it is possible to apply electrospray to the analysis of these macromolecules, because a molecule with a mass of 20,000 daltons that carries 25 charges will yield an ion observable at 800 daltons.

Software algorithms can be used to derive the original molecular weight from the observed ion peaks.

Smaller molecules yielding molecular ions also benefit from electrospray because the improved efficiencies inherent at atmospheric pressure ionization lead to greater sensitivity than can be achieved with conventional LC-MS interfaces.

In addition, the design of the electrospray ion source can permit a molecular ion to undergo a process known as collisionally induced decomposition.

In this process, the ion collides with neutral gas molecules and then fragments to yield smaller ions whose masses depend on the structure of the original molecular ion. Thus, structural information as well as the molecular weight of an unknown compound may be obtained.

In practical use, the effluent stream of a conventional HPLC is split prior to entering the electrospray source to provide the low flow rates required for good spraying.

To avoid splitting, auxiliary spraying techniques such as pneumatic or ultrasonic nebulizers have been added to the basic electrospray configuration, although some decrease in sensitivity is generally observed at these higher flow rates.

—Alexander Schilling

Alexander Schilling is an applications engineer for Hewlett-Packard Co., Naperville, IL.

44 R&D Magazine, February 1993

Preparation of dinitrile 2 via cyanoethylation:

Freshly distilled benzylamine (1) (10.72 g, (100 mmol) was dissolved in acrylonitrile (500 mL) at room temperature. Glacial acetic acid (11.5 mL, 200 mmol)

was added, and the resulting mixture was heated at reflux for 24 hours. The mixture was then cooled to room temperature, and excess acrylonitrile was removed under reduced pressure. The residue was partitioned between methylene chloride (250 mL) and concentrated ammonia solution (100 mL). The separated organic phase was washed once with water and dried with sodium sulfate. Concentration under vacuum afforded 19.34 g (91% yield) of dinitrile <u>2</u> as a slightly yellow oil.

^{1}H NMR (300 MHz): δ 7.34 (m, 5 H), 3.71 (s, 2 H), 2.90 (t, 4 H), 2.44 (t, 4 H).

^{13}C NMR (75 MHz): δ 137.50, 128.57, 128.51, 118.42, 58.15, 49.50, 16.77.

MS: m/*e* 236 (M + Na^+), 214 (M + H^+)

Preparation of primary amine 3 via reduction with diisobutylaluminum hydride:

The dinitrile 2 (1.60 g, 7.50 mmol) was dissolved in anhydrous tetrahydrofuran (300 mL) in a flame-dried three-neck flask. Diisobutylaluminum hydride (50 mL of a 1.5 M solution in toluene, 75 mmol) was added dropwise over 70 minutes. The mixture was then heated at reflux for 24 hours. After the mixture was cooled to room temperature, methanol (15 mL) was added dropwise to the vigorously stirred mixture. After a further 20 minutes of stirring, the formed white precipitate was removed by vacuum filtration and rinsed several times with methanol. The combined filtrate and washings were concentrated under vacuum to yield a clear gel. Acetonitrile (30 mL) was added, and the gel was broken up with a spatula. The cloudy mixture was vacuum-filtered and the solid, precipitate was washed several times with acetonitrile. The filtrate and washings were concentrated under reduced pressure to give 1.40 g (84% yield) of amine 3 as a clear oil.

^{1}H NMR (300 MHz): δ 7.31 (m, 5 H), 3.53 (s, 2 H), 2.71 (t, 2 H), 2.46 (t, 2 H), 1.62 (m, 2 H).

^{13}C NMR (75 MHz): δ 139.88, 128.80, 128.19, 126.82, 58.81, 51.39, 40.37, 30.31.

MS: *m/e* 222 (M + H^+),165.

Preparation of tetranitrile 4 via cyanoethylation:

Amine 3 (0.167 g; 0.755 mmol) was dissolved in acrylonitrile (10 mL) at room temperature. Glacial acetic acid (0.173 mL, 3.02 mmol) was added, and the mixture was heated at reflux for 24 hours. The mixture was then cooled to room temperature, and excess acrylonitrile was removed under reduced pressure. The residue was partitioned between chloroform (30 mL) and concentrated ammonia solution (10 mL). The separated organic phase was washed once with water and dried with sodium sulfate. Concentration under vacuum afforded a yellowish oil, which was purified by chromatography on 20 g of neutral alumina (elution with 150 mL of 2:1 methylene chloride/ethyl acetate, followed by 100 mL of 1:1 methylene chloride/ethyl acetate and 50 mL of 49:49:1 methylene chloride/ethyl acetate/methanol) to give 0.132 g (40% yield) of a slightly yellow oil.

^{1}H NMR (300 MHz): δ 7.31 (m, 5 H), 3.55 (s, 2H), 2.79 (t, 8 H), 2.51 (m, 8 H), 2.41 (t, 8 H), 1.61 (m, 4 H).

^{13}C NMR (75 MHz): δ 139.64, 128.80, 128.24, 126.97, 118.65, 58.85, 51.85, 51.36, 49.55, 25.18, 16.86.

MS: *m/e* 456 (M + Na^+),434 (M + H^+).

Preparation of primary amine 5 via reduction with diisobutylaluminum hydride:

The tetranitrile 4 (0.104 g, 0.240 mmol) was dissolved in anhydrous tetrahydrofuran (15 mL) in a flame-dried two-neck flask. Diisobutylaluminum hydride (3.2 mL of a 1.5 M solution in toluene, 4.80 mmol) was added dropwise over 20 minutes. The solution was then heated at reflux for 24 hours. After the solution was cooled to room temperature, methanol (3 mL) was added dropwise to the vigorously stirred mixture. After a further 30 minutes of stirring, the formed white precipitate was removed by

vacuum filtration and rinsed several times with methanol. The combined filtrate and washings were concentrated under vacuum to produce a clear gel. Methylene chloride (3 mL) was added, and the resulting cloudy solution was again filtered. The filtrate was concentrated under reduced pressure to give 0.0627 g (55% yield) of a clear oil.

^{1}**H NMR** (300 MHz): The spectrum was quite complex, but contained large signals in the regions δ 1.4-1.8 (aliphatic H), δ 2.2-2.8 ($R_2NC\underline{H_2}R$) δ 3.4-3.6 ($PhC\underline{H_2}NR_2$) and δ 7.2-7.4 (aromatic H).

^{13}**C NMR** (75 MHz): The spectrum was quite complex, but contained strong signals in the regions δ 28-34 ($R_2NCH_2\underline{C}H_2CH_2NR_2$), δ 40-60 ($R_2N\underline{C}H_2R$) and δ 124-142 (aromatic C)

MS: *m/e* 450 (M + H^+), 433, 393, 335.

Discussion and Results

The cyanoethylation of benzylamine (1) to produce the dinitrile **2** (See Scheme I) was carried out using the procedure of Vögtle et al. (Buhleier, E.; Vögtle, F. et al. *Synthesis* **155** (1978), copy attached, Vögtle 1978 article). This method gave the product with good yield and purity without chromatographic purification.

The dinitrile **2** was reduced to the branched primary amine compound **3** using diisobutylaluminum hydride (DIBAH) as described in the recent paper by Moors and Vögtle (Moors, R.; Vögtle, F. *Chem Ber.* **126,** 2133 (1993), copy attached, Vögtle 1993 article). This procedure gave a higher yield and greater purity (as judged by ^{1}H and ^{13}C NMR and the mass spectra [see Fig. I]) of the product compared to the product obtained using the sodium borohydride/cobalt (II) chloride as the reducing agent (see Vögtle 1978 article, *supra*). Some of the impurities were common to both procedures. However, there was still a significant amount of contaminants present. It was also

Scheme I: Synthesis of Branched Amine **5**

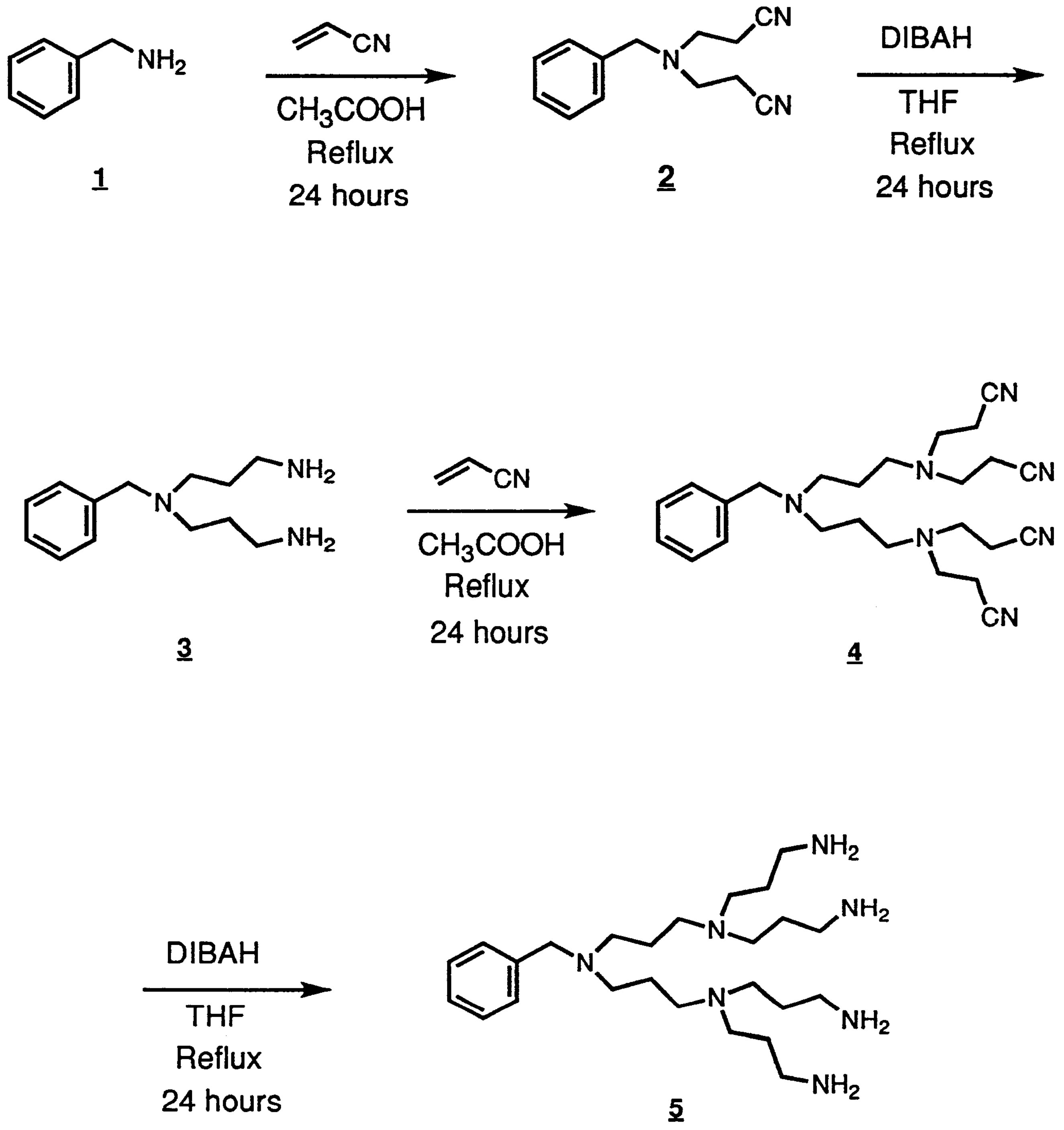

Figure 1. Electrospray Mass Spectra (MS) and ^{13}C NMR Spectra for Branched Amine 3 as Prepared Using *(i)* DIBAH or *(ii)* $NaBH_4/Co(II)Cl_2$ as Reductant.

(i) MS of DIBAH Product

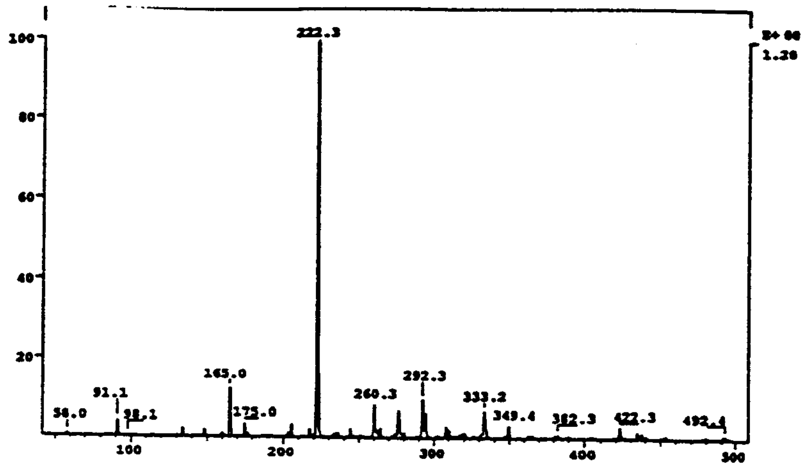

(ii) MS of $NABH_4/Co(II)Cl_2$ Product

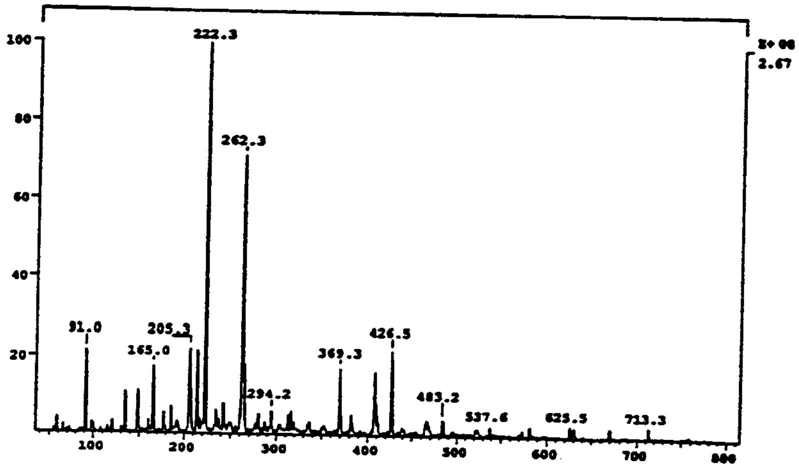

(i) ^{13}C NMR of DIBAH Product

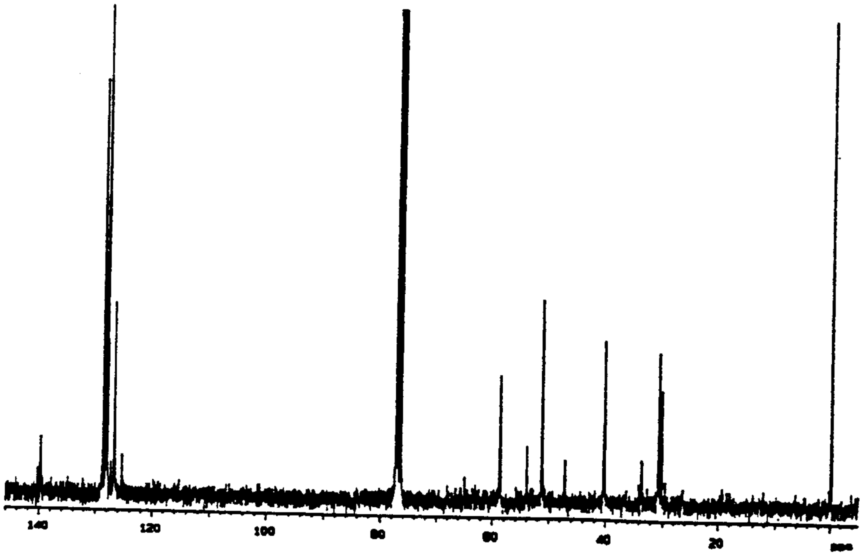

i) ^{13}C NMR of $NaBH_4/Co(II)Cl_2$ Product

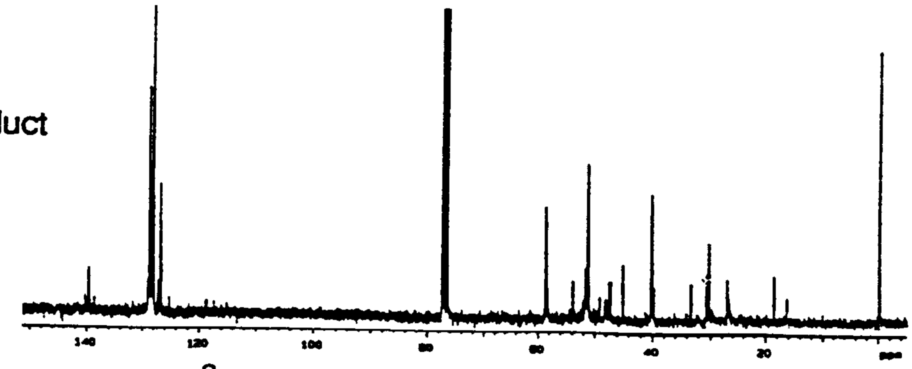

difficult to remove all of the solid aluminum salt by-products from the product, which necessitated an additional filtration step in the procedure.

(Note, however, that this diisobutylaluminum hydride reduction process was not known or available as of the priority or filing date of the present application.)

In the mass spectrum of the amine **3** product, the main contaminant (at m/*e* 165) has a molecular weight that is 57 less than the expected product. This contaminant is very likely to be the retro-Michael addition product **6** (See Fig. 2). I was not able to remove this contaminant by chromatographic means, so the impure product was used directly in the next reaction.

Fig 2: Retro-Michael addition product **6**.

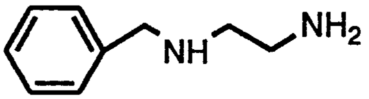

6

The cyanoethylation of primary amine **3** to produce the tetranitrile **4** (see Scheme 1) was also performed using the procedure described in the Vögtle 1978 publication (*supra*). The product was much less polar that the amine **3**, which allowed the removal of contaminating side-products by employing chromatography on alumina. As expected, the main isolated side-product resulted from cyanoethylation of the retro-Michael addition product **6**, trinitrile **7** (see Fig. 3). Spectra for the two purified compounds, tetranitrile **4** and side product **7**, are shown in Fig. 4.

Figure 4. Electrospray Mass Spectra (MS) and ^{13}C NMR Spectra for (i) Side-Product 6 and (ii) Tetranitrile 4

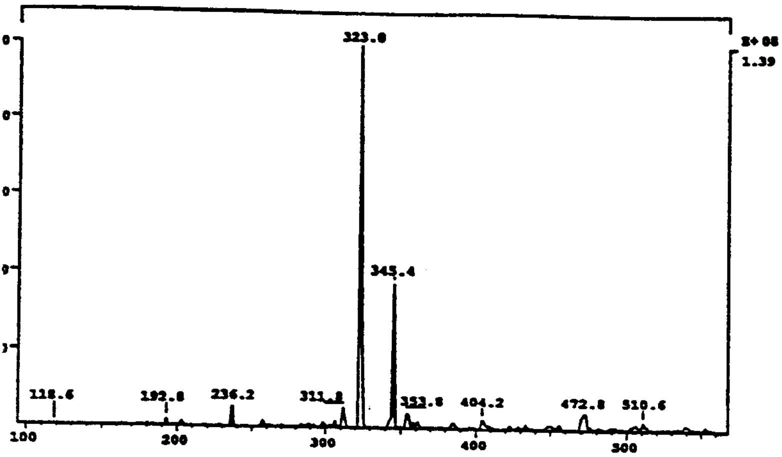

(i) MS of Side Product 6

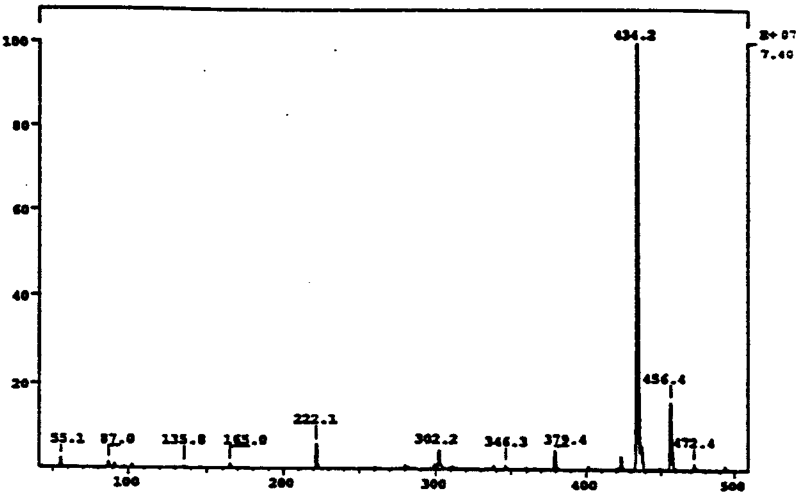

(ii) MS of Tetranitrile 4

(i) ^{13}C NMR of Side Product 6

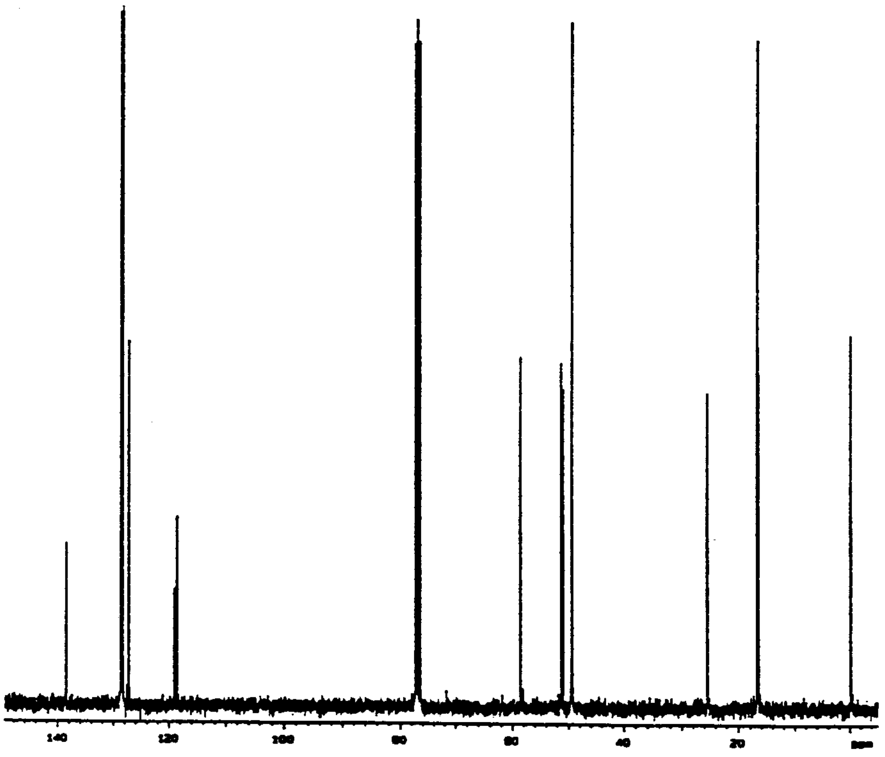

(ii) ^{13}C NMR of Tetranitrile 4

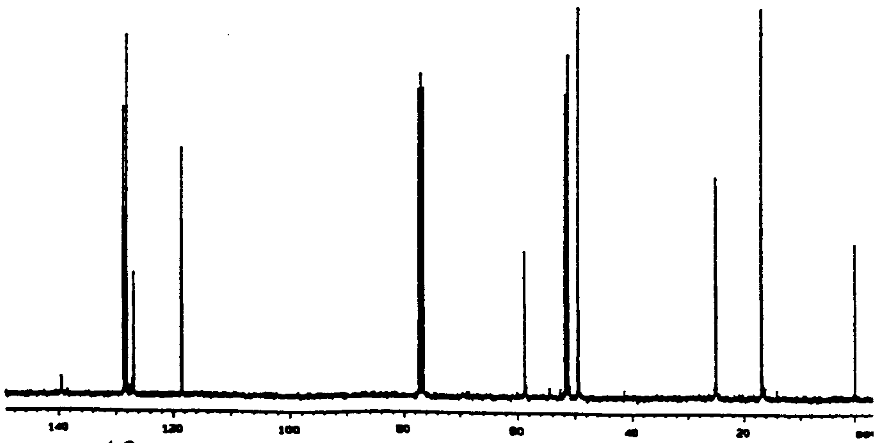

Fig. 3: Cyanoethylated side product.

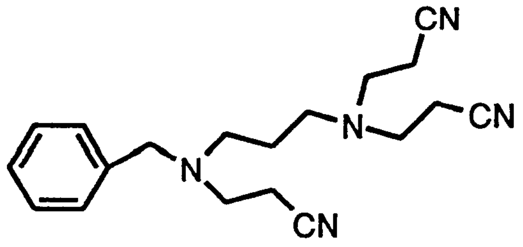

7

The purified tetranitrile 4 was reduced to the branched primary amine 5 (See Scheme I) using the same DIBAH procedure (Vögtle 1993 article, *supra*) as was employed to prepare primary amine 3. Although a mixture of different products was obtained, the electrospray mass spectrum showed three main peaks, one of which (M/*e* 449) is correct for primary amine 5 (see Fig. 5). Note that this mass spectrum is the derived spectrum, which gives the M/z mass values). The other two peaks (M/*e* 392 and 335) are correct for the retro-Michael addition product 8 (loss of one ethylamine branch) and double retro Michael addition products 9 and 10 (loss of two ethylamine branches; see Fig. 6 for structures). These retro-Michael addition products are expected, given that a retro-Michael addition side-product (6) is produced in the corresponding DIBAH reduction of dinitrile 2. The retro-Michael product is most likely produced by the extensive heating (66°C, 24 hours) of the reaction mixture in the reduction procedure.

Figure 5. Electrospray Mass Spectrum (MS), and ^{13}C and ^{1}H NMR Spectra for the Product Obtained from the DIBAH Reduction of Tetranitrile **4**.

(i) Mass Spectrum

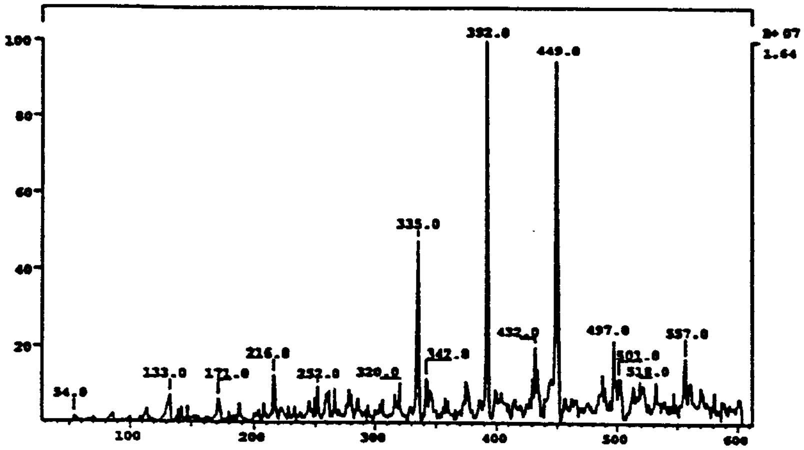

(ii) ^{13}C NMR

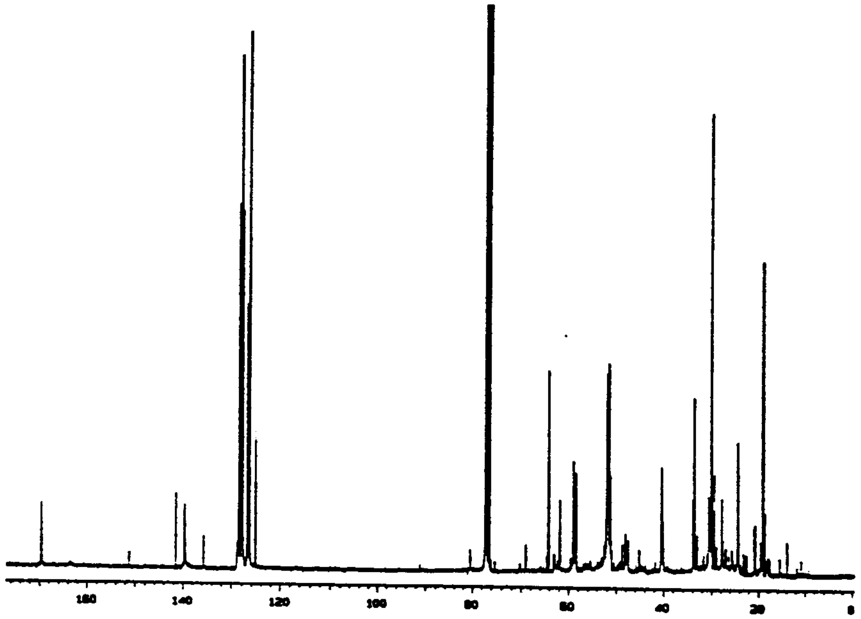

(iii) ^{1}H NMR

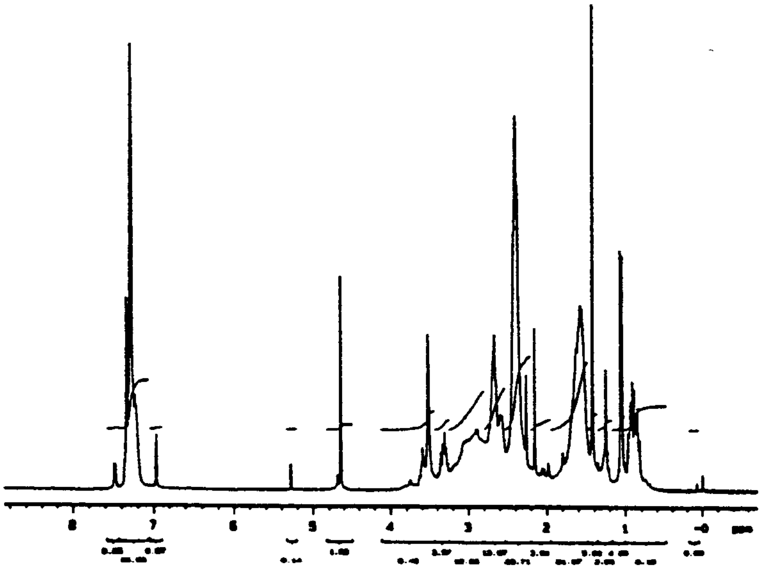

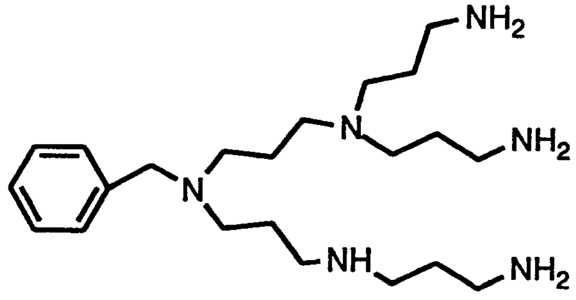

8

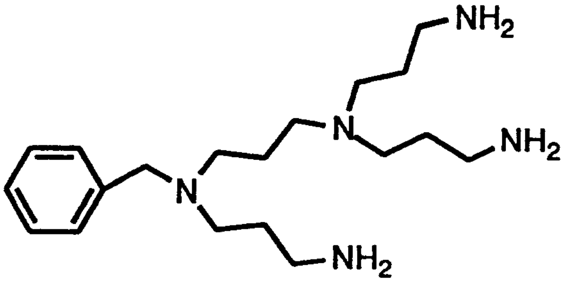

9

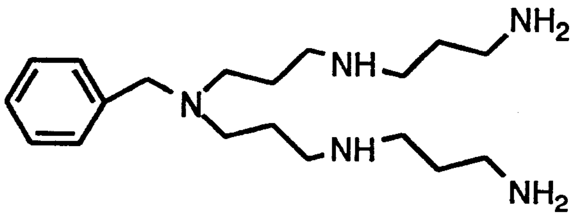

10

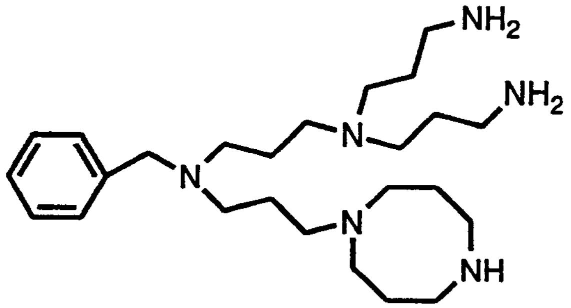

11

Fig. 6: Defect Structures Produced by the DIBAH Reduction of Tetranitrile 4.

A small peak is seen at 432, corresponding to the loss of 17 in molecular weight from the primary amine 5. This peak is most probably due to the formation of secondary amine 11, a common type of side-product in the reduction of nitriles (see Christoph Rickert's Declaration for a discussion of this side-reaction).

Although the ^{1}H and ^{13}C NMR spectra of the product are quite complex as a result of the presence of the defect compounds (See Fig. 5), they are consistent with a mixture of amines 5 and 8 - 11. These results indicate that when the product amine 5

is successfully produced, its presence can be easily verified by electrospray mass spectroscopy. It should be noted that when tetranitrile 4 is reduced with $NaBH_4/Co(II)Cl_2$ (as described in the Vögtle 1978 publication, *supra*), no branched amine 5 can be detected by electrospray mass spectroscopy (these experiments were performed by Christoph Rickert of MMI).

(It is important to note again that the diisobutylaluminum hydride reduction process described in the Vögtle 1993 publication (*supra*) was not known or available as of the priority or filing date of the present application.)

THAT, the recently-published DIBAH nitrile reduction procedure of Vögtle (Vögtle 1993 article, *supra*) can be successfully employed to produce branched polyamines (represented here by amine 5), albeit in low yield and purity. In contrast, use of the $NaBH_4/Co(II)Cl_2$ nitrile reduction procedure described in the older Vögtle publication (Vögtle 1978 article, *supra*) gave no detectable amount of amine 5 (this experiment was carried out by Christoph Rickert of MMI and is discussed in his Declaration). For these reasons, the compounds now claimed by the present Japanese patent application could not have been known or prepared by the Vögtle 1978 article process (*supra*).

The undersigned DECLARANT, Lars Piehler declares further that all statements made herein of his own knowledge are true and that all statements made on information and belief are believed to be true; and further that these statements were made with the knowledge that willful false statements and the like so made are

punishable by fine or imprisonment, or both, under Section 1001 of Title 18 of the United States Code.

Date: 12/10/1993

Lars Piehler

Lars T. Piehler, Ph.D.
